# Changes in self-reported sleep duration with age - a 36-year longitudinal study of Finnish adults

**DOI:** 10.1186/s12889-020-09376-z

**Published:** 2020-09-09

**Authors:** Christer Hublin, Lassi Haasio, Jaakko Kaprio

**Affiliations:** 1 Finnish Institute of Occupational Health, University of Helsinki, Helsinki, Finland; 2 Department of Public Health, University of Helsinki, Helsinki, Finland; 3 Institute for Molecular Medicine Finland FIMM, University of Helsinki, PO Box 20 (Tukholmankatu 8B), Helsinki, Finland

**Keywords:** Sleep length, Population, Longitudinal study, Weibull regression models, Sleep ontogeny

## Abstract

**Background:**

Sleep deprivation is often claimed to be increasingly common, but most studies show small changes in sleep duration over the last decades. Our aim was to analyze long-term patterns in self-reported sleep duration in a population-based cohort.

**Methods:**

Members of the Older Finnish Twin Cohort have responded to questionnaires in 1975 (*N* = 30,915 individuals, response rate 89%, mean age 36 years), 1981 (24,535, 84%, 41 years), 1990 (12,450, 77%, 44 years), and 2011 (8334, 72%, 60 years). Weibull regression models were used to model the effects of follow-up time and age simultaneously.

**Results:**

Sleep duration has decreased in all adult age groups and in both genders. The mean duration was in men 7.57 h in 1975 and 7.39 in 2011, and in women 7.69 and 7.37, respectively. The decrease was about 0.5 min in men and 0.9 in women per year of follow-up. In the age-group 18–34 years, mean sleep length was 7.69 h in 1975 and 7.53 in 1990. Among 35–54-year-old it was 7.57 h in 1975 and 7.34 in 2011, and in the age group of 55+ year olds 7.52 and 7.38, correspondingly. The change was largest in middle-aged group: about 23 min or about 0.6 min per year of follow-up.

**Conclusions:**

There has been a slight decrease in mean sleep duration during the 36-year follow-up. Although the sleep duration was longer in 1970s and 1980s, the probable main cause for the change in this study population is the effect of aging.

## Background

Good sleep is fundamental for health. Chronically deficient sleep, manifested as decreased amount or quality of sleep or mistiming of sleep, is associated with many negative health outcomes [1]. Human physiology undergoes many age-related changes across the lifespan. With age, sleep becomes shorter, lighter and more easily disturbed by both external and internal factors. Generally, these trends are most evident on group level, with considerable individual variation in all ages [2, 3].

Many factors affect sleep length. In an earlier study we investigated the contribution of genetic factors to stability and change of sleep length among adults [4]. The effect was modest (proportion of variance accounted for by inter-individual differences in genetics was about 0.3) but stable (genetic correlation 0.76 over a 15-year study period), indicating that mostly the same genes are active throughout adulthood. While environmental effects had a major effect at each assessment time, the environmental effects were only modestly correlated over time, which indicates that external effects are important but mostly short-lived. In another population-based Finnish study most important determinants of short and long sleep duration were gender, physical tiredness, sleep problems, marital status, main occupation, and physical activity [5]. However, they only accounted for 16% of the variance of sleep duration, suggesting a multifactorial and polygenic aetiology to sleep duration.

It is a common view that sleep deprivation has grown into an epidemic in modern societies [6, 7]. Indeed, a systematic review with data from 1905 to 2008 and 20 countries on children and adolescents aged 5–18 years indicated a decrease of more than 1 h per night in sleep length in general, although some countries showed increases [8]. However, in adults the scientific evidence of a similar development is far from unambiguous [9]. Some US studies have reported decreased average sleep duration and/or increase in proportion of those with short (< 6 h) sleep [10, 11] but other have not found meaningful changes [12] or even contrary changes [13–15]. Moreover, the trend seems to vary from country to country. In a recent review of studies from 12 countries sleep duration had increased in four countries, decreased in three countries, and showed no clear change in four countries, and the results were conflicting in the United States [16]. The changes were quite small, around a minute per night each year from 1960s until the 2012. A recent review of 168 studies with objectively recorded (polysomnography or actigraphy) sleep duration over the last 50+ years showed no significant association of duration with study year [17]. It can be concluded that these results on both subjective and objective sleep duration challenge the notion of a modern epidemic of insufficient sleep in adults.

The aim of this study was to describe and analyze long-term patterns in self-reported sleep duration, using four time points of measurement in a population-based adult cohort over a 36-year follow-up period.

## Methods

### Population sample

The Older Finnish Twin Cohort includes twins born in Finland before 1958 where both twins in a pair are alive in 1975 [18, 19]. As these pairs of the same gender were selected from the Central Population Registry of Finland in 1974, the Cohort is population-based and its overall mortality and cancer incidence does not differ from that of the general population [18, 20]. Cohort members have responded to four mailed questionnaires in which sleep duration was asked. In 1975, the first survey had a response rate was 89% (*N* = 30,915 twin and non-twin individuals with valid responses to sleep length). In 1981, the second wave survey had a a response rate of 84% (*N* = 24,535 twin individuals). In 1990, we restricted the survey to younger pairs, who were born in 1930–1957 and where both co-twins were resident in Finland in 1987. The response rate was 77%, (*N* = 12,450 twin individuals). For the fourth survey in 2011/2012, we approached all twins in the cohort who were alive and resident in Finland, and who were born in 1945–1957. The response rate was 72% (*N* = 8334 twin individuals). The questionnaires included about 100 questions on demographic, social, health/illness and lifestyle variables. The number of sleep-related variables in the different questionnaires varied, but sleep length was elicited in all four questionnaires [4, 21, 22]. See supplementary files for English translations.

**Fig. 1:**
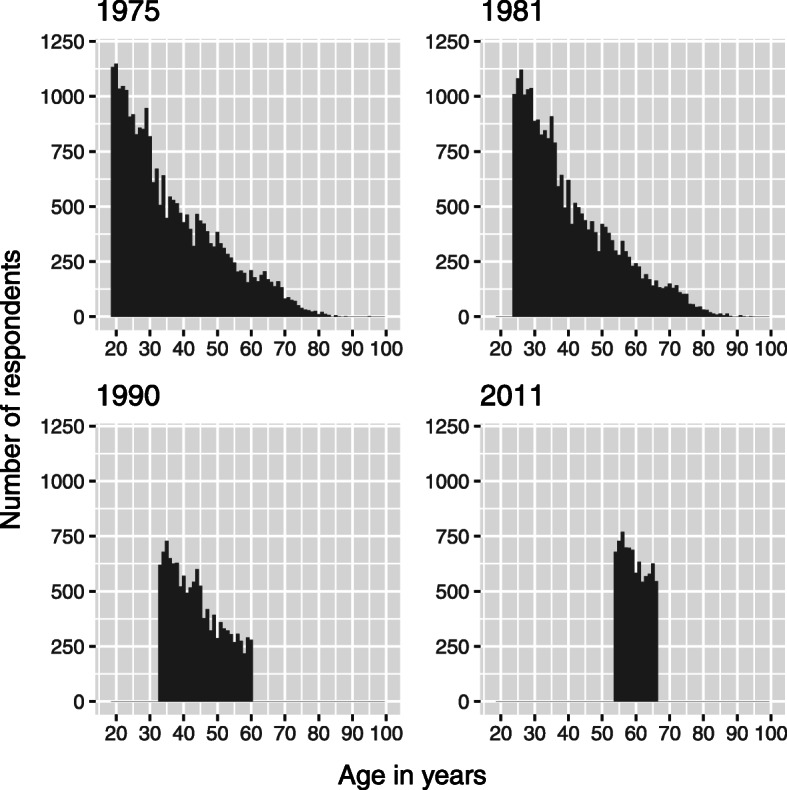
Age distributions of the respondents in each of the four points of measurement. X-axis: age in years. Y-axis: number of respondents

Characteristics of subjects included in the present study at each of the four measurement points are given in Table 1. Age distributions of the respondents in each of the four points of measurement are given in Fig. 1.

**Table 1:** Characteristics of subjects included in the present study at each of the four measurement points. Persons with missing data on sleep length are excluded

Year of questionnaire	Number of individuals	Women (%)	Age (mean ± 1.96 x Standard deviation) in years Range of age (birth years)
1975	30,915	51.4	35.7 ± 14.6 18–95 (1880–1957)
1981	24,535	51.7	40.5 ± 13.7 24–101 (1880–1957)
1990	12,450	54.4	43.9 ± 7.8 33–60 (1930–1957)
2011	8334	55.4	59.7 ± 3.7 54–66 (1945–1957)

### Sleep length data

Sleep length was queried as follows “How many hours do you usually sleep per 24 hours?” In the first survey in1975 we gave seven response alternatives (less than 4 h, 5, 6, 7, 8, 9, and 10 h or more). For the three later questionnaires in 1981, 1990 and 2011, we used each time the same nine alternatives (6 h or less, 6.5, 7, 7.5, 8, 8.5, 9, 9.5, and 10 h or more). The four questionnaires used in the study are appended as supplementary material (Additional files 1, 2, 3 and 4).

### Statistical methods

Standard statistical methods were used in data analysis to obtain descriptive statistics such as frequencies and proportions when comparing sleep duration groups and implemented in the Stata package (version 13.1, Stata Corp, College Station, Texas, United States of America, www.stata.com). In this study self-report of sleep duration is the dependent variable, and as explanatory variables we used birth cohort, age of the respondent at each measurement, and gender.

**Fig. 2:**
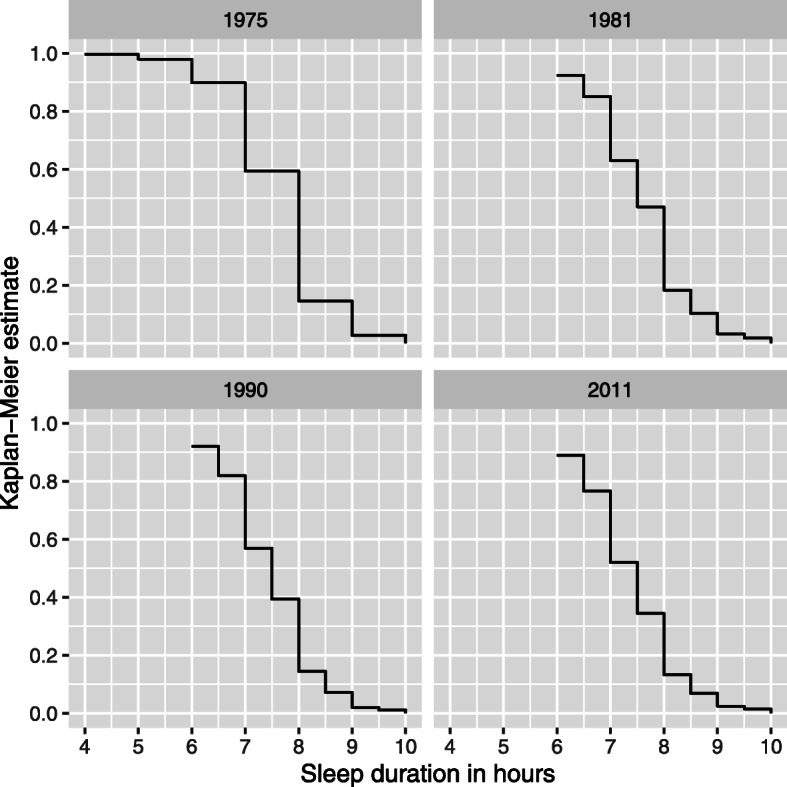
Kaplan-Meier estimates of sleep duration in the four points of measurement. X-axis: sleep duration in hours. Y-axis: proportion of respondents with a certain sleep duration; e.g. in 1975 90% of the respondents slept 6 h or more, and 60% 7 h or more

The mathematical model treated transitions from one category to the next as interval censored data, so that the interval was larger in 1975 (one hour) compared to half-an-hour in the other three surveys. A survival model was then applied using a likelihood function and a Weibull regression model. The model assumes that there is an underlying continuous distribution of sleep length, and the reported sleep length are marks in the distribution that are used to model the latent continuous variable. Figure 2 shows the sleep durations as Kaplan-Meier curves; these are then modelled.

We used a Weibull regression model as a parametric regression model for survival data [23]. Because the data was composed of repeated measurements on the same persons, we modelled the covariance structures using random effects. We further considered the lack of statistical independence within twin pairs due to sampling of twin pair candidates in 1975 by modelling the twin pairs as random effects. Finally, we did not have sleep length as a continuous measure (e.g. hours and minutes) but rather as time intervals, so interval censoring was used [24]. The detailed statistical approaches are described in Haasio [25].

## Results

Distributions of responses by gender and age-group in each questionnaire are given in Table 2. Generally, the respondents have reported slightly longer sleep duration in the earlier than in the later questionnaires. This is seen in both genders: the mean sleep duration was in 1975 in men 7.57 h and in women 7.69 h, and in 2011 7.39 and 7.37 h, respectively. This means a decrease of about 0.5 min in men and 0.9 in women per year of follow-up.

**Table 2:** Sleep duration (hours; mean, standard deviation) in each of the four questionnaires

Year of questionnaire	Men	Women	Age group 18–34 years	Age group 35–54 years	Age group 55+ years
1975	7.57, 0.95	7.69, 0.98	7.69, 0.92	7.57, 0.93	7.52, 1.20
1981	7.53, 0.85	7.67, 0.89	7.63, 0.83	7.56, 0.84	7.64, 1.06
1990	7.39, 0.81	7.55, 0.84	7.53, 0.83	7.46, 0.83	7.50, 0.88
2011	7.39, 0.89	7.37, 0.86	No data	7.34, 0.84	7.38, 0.88

There is a similar change also when assessing the three age-groups of similar age range in different years of questionnaires. For example, in the age group 35–54 years the mean sleep duration was 7.57 h in 1975 and 7.34 in 2011, giving a decrease of 0.6 min per year of follow-up. However, when assessing the different age groups within each year of questionnaire the change is less consistent: when comparing the youngest group (18–34 years) to the oldest group (55+ years), there was some decrease in mean sleep duration in 1975 (from 7.69 to 7.52 h), but no clear change in the other three survey years.

The regression modelling results showed both age and cohort effects in sleep duration (Table 3). Going from the age group 18–34 years to age group 35–54 years or to 55+ years shortens sleep duration; it can be noted that the hazard ratios for the two older age-groups are very similar and overlap. The difference with respect to birth cohort, those born in 1920–39 and those born in 1940–57, slept less than the reference group (those born in 1880–99). On the other hand, those born in 1900–19 did not sleep statistically significantly less than those born in 1880–99.

**Table 3:** The hazard ratios and the confidence intervals of the fitted model for shorter sleep. The effect of the birth cohorts is compared to those born in 1880–1899. The effect of age group is compared to those being 18–34 years old. Male gender is compared to females

	Hazard ratio (95% CI)
Birth cohort < 1900	1.00
Birth cohort 1900–1919	1.11 (0.96 to 1.30)
Birth cohort 1920–1939	1.41 (1.21 to 1.64)
Birth cohort 1940–1957	1.62 (1.40 to 1.89)
Age group 18–34 years	1.00
Age group 35–54 years	1.24 (1.21 to 1.28)
Age group 55+ years	1.23 (1.19 to 1.27)
Female gender	1.00
Male gender	1.13 (1.10 to 1.15)

These results suggest that the sleeping habits may have changed modestly over time, and the decrease in the sleep duration may not solely be explained by aging of the population. In other words, the younger generations sleep less when comparing the sleep duration of the same age groups belonging to the different birth cohorts. Overall, when adjusted for age and birth cohort, men sleep less than women.

**Fig. 3:**
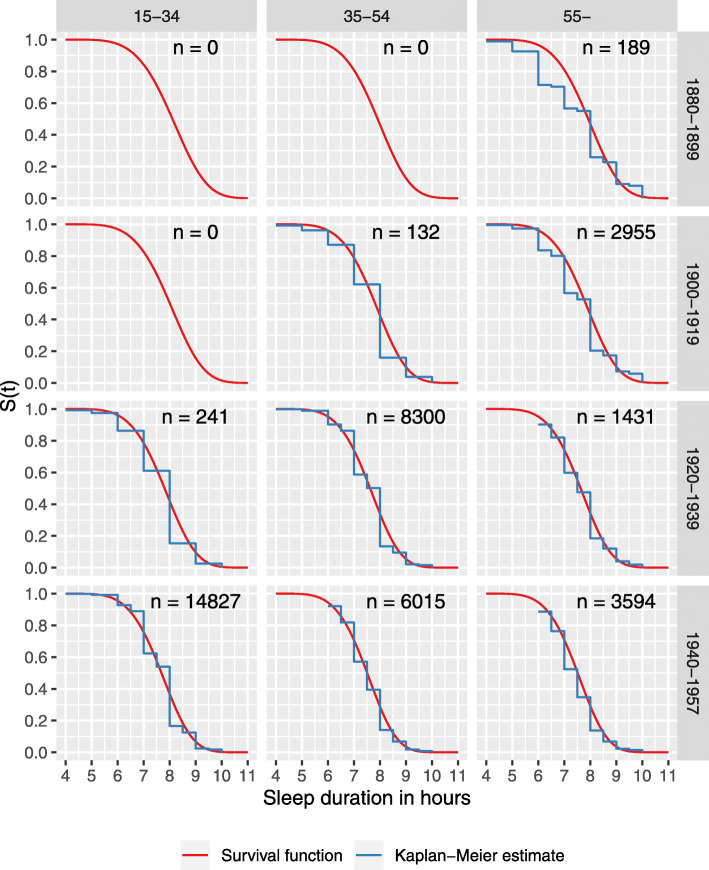
Survival functions and corresponding Kaplan-Meier estimates for each age group and birth cohort calculated using a fitted regression model. The number of observations given in each cell. The total number of observations is larger than the number of participants as there are one to four observations of each participant

The changes are also seen in Fig. 3. In 1975 and in 1981 about 60% of the respondents slept 7 h or more, but in 1990 less than 60% and in 2011 only a bit more than 50% did so. Sleep duration decreases with age but only when young adults are compared to middle-aged. There is no clear difference between middle-aged and those aged 55 years or more. This trend may not be, however, explained only by aging alone as there also is a cohort effect, those born earlier slept more at the same age than those born later (Fig. 3).

## Discussion

The main result of the present study was that the mean sleep duration slightly decreased in the adult study population over the 36-year follow-up. The change was seen in all adult age groups and in both genders. Overall the changes are quite small. The mean decrease in men was about 18 min and in women about 32 min (about 0.5 and 0.9 min per year of follow-up, respectively). In age-groups the largest change was in middle-aged persons: about 23 min or 0.6 min per year of follow-up. Although the sleep duration was longer in the earlier years, the probable main cause for the change in this study population is the effect of aging.

In a systematic review on secular trends on sleep duration from 15 countries from the 1960s until the 2000s showed also small changes [26]. Self-reported average sleep duration of adults had increased in 7 countries (from 0.1 to 1.7 min per night each year, e.g. Canada and Britain) and decreased in 6 countries (from 0.1 to 0.6 min per night each year, e.g. Finland and Germany). The Finnish data was based on reanalysis of all available data from surveys carried out from 1972 to 2005, including about 440,000 self-reports of sleep duration [27], to which the present study contributed data from the first three surveys (1975, 1981, and 1990). The decrease in Finland was 18 min during a period of 33 years or about 0.5 min per year of follow-up. In all, the present results are well in accordance with the results from previous studies both from Finland and other countries. It has been pointed out that there is no validated measure of self-reported sleep duration and the different ways of asking (for example rounding sleep to within 15 or even 60 min) may affect the estimate [9]. As the changes in each country and the differences between the countries are small, it is possible that they are at least partly due to methodological causes.

The strong association between health and sleep has also practical implications. It has long been known that many patients with somatic or psychiatric disorders also suffer from impaired sleep. Not until the last decades have we learned that poor sleep is not only comorbid with many disorders, but may also be a contributing causative factor, as shown in, for example, cardiovascular disease [1, 28–30]. Evidence for a causal role in breast cancer [31] and type 2 diabetes [32, 33] is less convincing. Genetic analyses suggest shared genetic liability to sleep duration and a variety of other medically relevant traits and diseases [34], but the causal paths require more studies. Thus, in clinical practice, it is important to evaluate sleep, as poor quality may increase the risk of many common diseases, decrease the quality of life, and impair patients’ treatment responses.

The present study has several strengths. First, the follow-up time of 36 years is exceptionally long. Second, the study cohort is population-based and therefore the results can probably be generalized to the population at large, at least in countries with a very high human development index. Compared to general population, the Twin Cohort shows no differences in e.g. overall mortality [18] or cancer incidence [20]. Sleep lengths in twins have been similar as in other Finnish studies [27]. In addition, our twin cohort has taken part in multiple genome-wide association studies, with cohort specific effect size estimates that do not differ from the meta-analysis results [35, 36]. The results are also valuable from an ontogeny point of view. Thirdly, longitudinal cohort studies may give a more reliable picture of age-related changes than a cross-sectional study or combinations of these, as suggested by our earlier 36-year follow-up study on the quality of sleep in the same cohort [37].

There are also some limitations that must be kept in mind. Information on sleep length is derived from self-reports. As we have discussed earlier [4], objective assessments of sleep length for thousands of participants three to four decades ago was logistically and even technologically not possible. Large-scale studies continue to rely on self-reported sleep length, even though it has less accuracy, which affects in particular those with fragmented sleep (e.g. insomniacs). The 1990 and 2011 questionnaires did not cover age groups 65+ years, and 2011 did not include subjects <55 years, thus the conclusions must be especially cautious regarding them. Because of the structure of the study population only limited conclusions of the possible intergenerational changes in sleep duration can be made. Our sample consists of twins but it is representative of general population. In our 1975 questionnaire we had both twins and persons who were not twins as a result of our ascertainment procedure of identifying as twins pairs of persons born on the same day, in the same local community, of the same sex and with the same surname at birth [38]. The twins and non-twins did not differ in sleep duration or sleep quality. We are not aware of studies indicating significant differences in sleep and its disorders between twin individuals and the rest of the general population. Additionally, we cannot rule out that some fraction of the apparently age-related changes is in fact due to contemporaneous secular trends, such as the 24/7 society and changes in working life including reduced physical strain and the increasing use of portable screen-based media devices. These trends probably affect more younger adults (< 35-year-old) who were not represented in the last survey made during time when the use of this technology rapidly increased. The three first surveys were made during the era with no smartphones and social media. Even at the time of the fourth survey, internet and social media use was much less than now, a decade later. The last wave was collected in 2011/2012 among older adults born 1945 to 1957, who have adopted smart phones and tablets at a slower rate than adolescents and young adults.

## Conclusions

Our results indicate no major changes in self-reported sleep duration in adults, similarly as in other population-based studies, and contrary to the common views often presented in media that serious sleep deprivation is a widespread problem. However, small decreases were seen in both genders and in all age-groups, in the range of tenths of a minute per year of follow-up as also in the earlier studies. Over time, sleep duration was decreased slightly more in women than in men, and in age-groups the largest change was in middle-aged persons. In all age-groups, the reported sleep duration was slightly longer in the earlier questionnaires than in the later questionnaires. In our opinion the probable main cause for these slight changes in this study population is the effect of aging.

## Supplementary information

**Additional file 1:.** FTC old cohort 1975 wave 1 questionnaire English translation. Unofficial translation of mailed questionnaire used in wave 1 of the older Finnish Twin Cohort in 1975, originals in Finnish and Swedish.

**Additional file 2:.** FTC old cohort 1981 wave 1 questionnaire English translation. Unofficial translation of mailed questionnaire used in wave 2 of the older Finnish Twin Cohort in 1981, originals in Finnish and Swedish.

**Additional file 3:.** FTC old cohort 1990 wave 3 questionnaire English translation. Unofficial translation of mailed questionnaire used in wave 3 of the older Finnish Twin Cohort in 1990, originals in Finnish and Swedish.

**Additional file 4:.** FTC old cohort 2011 wave 4 questionnaire English translation. Unofficial translation of mailed questionnaire used in wave 4 of the older Finnish Twin Cohort in 2011, originals in Finnish and Swedish.

## Data Availability

Persons interested in accessing the data should contact the corresponding author.

## References

[1] Luyster FS, Strollo PJ, Zee PC, Walsh JK (2012). Boards of directors of the American Academy of sleep medicine and the Sleep Research Society. Sleep: a health imperative. Sleep..

[2] Partinen M, Hublin C. Epidemiology of sleep disorders. In: Kryger M, Roth T, Dement WC, editors. Principles and practice of sleep medicine. St.Louis: Elsevier Saunders; 2011. p. 694–715.

[3] Vitiello MV (2006). Sleep in normal aging. Sleep Med Clin.

[4] Hublin C, Partinen M, Koskenvuo M, Kaprio J (2013). Genetic factors in evolution of sleep length – a longitudinal twin study in Finnish adults. J Sleep Res.

[5] Kronholm E, Härmä M, Hublin C, Aro AR, Partonen T (2006). Self-reported sleep duration in Finnish general population. J Sleep Res.

[6] Roenneberg T (2013). Chronobiology: the human sleep project. Nature..

[7] Van Cauter E, Knutson KL (2008). Sleep and the epidemic of obesity in children and adults. Eur J Endocrinol.

[8] Matricciani L, Olds T, Petkov J (2012). In search of lost sleep: secular trends in the sleep time of school-aged children and adolescents. Sleep Med Rev.

[9] Matricciani L, Bin YS, Lallukka T, Kronholm E, Dumuid D, Paquet C, Olds T (2017). Past, present, and future: trends in sleep duration and implications for public health. Sleep Health.

[10] Morbidity and Mortality Weekly Report (2005). National Center for Health Statistics Quick-Stats: Percentage of adults who reported an average of ≤ 6 hours of sleep per 24-hour period, by sex and age group—United States, 1985 and 2004.

[11] Ford ES, Cunningham TJ, Croft JB (2015). Trends in self-reported sleep duration among US adults from 1985 to 2012. Sleep..

[12] Knutson KL, Van Cauter E, Rathouz PJ, DeLeire T, Lauderdale DS (2010). Trends in the prevalence of short sleepers in the USA: 1975-2006. Sleep..

[13] Bin YS, Marshall NS, Glozier N (2013). Sleeping at the limits: the changing prevalence of short and long sleep durations in 10 countries. Am J Epidemiol.

[14] Basner M, Dinges DF. Sleep duration in the United States 2003-2016: first signs of success in the fight against sleep deficiency? Sleep. 2018;41. 10.1093/sleep/zsy012. 10.1093/sleep/zsy012 29325164

[15] Lamote de Grignon Pérez J, Gershuny J, Foster R, De Vos M (2019). Sleep differences in the UK between 1974 and 2015: Insights from detailed time diaries. J Sleep Res.

[16] Hoyos C, Glozier N, Marshall NS (2015). Recent evidence on worldwide trends on sleep duration. Curr Sleep Med Rep.

[17] Youngstedt SD, Goff EE, Reynolds AM, Kripke DF, Irwin MR, Bootzin RR, Khan N, Jean-Louis G (2016). Has adult sleep duration declined over the last 50+ years?. Sleep Med Rev.

[18] Kaprio J (2013). The Finnish twin cohort study: an update. Twin Res Human Genet.

[19] Kaprio J, Bollepalli S, Buchwald J, Iso-Markku P, Korhonen T, Kovanen V, Kujala U, Laakkonen EK, Latvala A, Leskinen T (2019). The older Finnish twin cohort - 45 years of follow-up. Twin Res Human Genet.

[20] Skytthe A, Harris JR, Czene K, Mucci L, Adami HO, Christensen K, Hjelmborg J, Holm NV, Nilsen TS, Kaprio J, Pukkala E (2019). Cancer incidence and mortality in 260,000 Nordic twins with 30,000 prospective cancers. Twin Res Human Genet.

[21] Paunio T, Korhonen T, Hublin C, Partinen M, Kivimäki M, Koskenvuo M, Kaprio J (2009). Longitudinal study on poor sleep and life dissatisfaction in a nationwide cohort of twins. Am J Epidemiol.

[22] Paunio T, Korhonen T, Hublin C, Partinen M, Koskenvuo K, Koskenvuo M, Kaprio J (2015). Poor sleep predicts symptoms of depression and disability retirement due to depression. J Affect Disord.

[23] Lee ET, Wang JW. Statistical methods for survival data analysis. Hoboken: Wiley series in probability and statistics; 2013.

[24] Kalbfleisch JD, Prentice RL (1980). The statistical analysis of failure time data.

[25] Haasio L. Suomalaisten unen pituuden muutoksen selittäminen ikä-kohortti-mallin avulla [explaining the change of duration in Finnish population using age-cohort-model (in Finnish)]. Master’s Thesis: Faculty of Science, University of Helsinki; 2016. http://urn.fi/URN:NBN:fi-fe2017112251682.

[26] Bin YS, Marshall NS, Glozier N (2012). Secular trends in adult sleep duration: a systematic review. Sleep Med Rev.

[27] Kronholm E, Partonen T, Laatikainen T, Peltonen M, Härmä M, Hublin C, Kaprio J, Aro A, Partinen M, Fogelholm M (2008). Trends in self-reported sleep duration and insomnia-related symptoms in Finland from 1972 to 2005: a comparative review and re-analysis of Finnish population samples. J Sleep Res.

[28] Liao LZ, Li WD, Liu Y, Li JP, Zhuang XD, Liao XX (2019). Causal assessment of sleep on coronary heart disease. Sleep Med.

[29] Daghlas I, Dashti HS, Lane J, Aragam KG, Rutter MK, Saxena R, Vetter C (2019). Sleep duration and myocardial infarction. J Am Coll Cardiol.

[30] Dashti HS, Jones SE, Wood AR, Lane JM, van Hees VT, Wang H, Rhodes JA, Song Y, Patel K, Anderson SG (2019). Genome-wide association study identifies genetic loci for self-reported habitual sleep duration supported by accelerometer-derived estimates. Nat Commun.

[31] Richmond RC, Anderson EL, Dashti HS, Jones SE, Lane JM, Strand LB, Brumpton B, Rutter MK, Wood AR, Straif K (2019). Investigating causal relations between sleep traits and risk of breast cancer in women: mendelian randomisation study. BMJ..

[32] Wang J, Kwok MK, Au Yeung SL, Li AM, Lam HS, Leung JYY, Hui LL, Leung GM, Schooling CM (2019). Sleep duration and risk of diabetes: observational and Mendelian randomization studies. Prev Med.

[33] Ferrie JE, Kivimäki M, Akbaraly TN, Tabak A, Abell J, Davey Smith G, Virtanen M, Kumari M, Shipley MJ (2015). Change in sleep duration and type 2 diabetes: the Whitehall II study. Diabetes Care.

[34] Dashti HS, Redline S, Saxena R (2019). Polygenic risk score identifies associations between sleep duration and diseases determined from an electronic medical record biobank. Sleep.

[35] Wang H, Lane JM, Jones SE (2019). Genome-wide association analysis of self-reported daytime sleepiness identifies 42 loci that suggest biological subtypes. Nat Commun.

[36] Ganna A, Ortega-Alonso A, Havulinna A, Salomaa V, Kaprio J, Pedersen NL, Sullivan PF, Ingelsson E, Hultman CM, Magnusson PK (2013). Utilizing twins as controls for non-twin case-materials in genome wide association studies. PLoS One.

[37] Hublin C, Lehtovirta M, Partinen M, Koskenvuo M, Kaprio J. Changes in sleep quality with age - a 36-year follow-up study of Finnish working-aged adults. J Sleep Res. 2018;27. 10.1111/jsr.12623. 10.1111/jsr.12623 29047168

[38] Kaprio J, Koskenvuo M (2002). Genetic and environmental factors in complex diseases: the older Finnish twin cohort. Twin Res.

