# Supplementary material for: Changes in self-reported sleep duration with age - a 36-year longitudinal study of Finnish adults
Source: BMC Public Health. 2020 Sep 9;20:1373. doi: 10.1186/s12889-020-09376-z (PMC7487757; doi:10.1186/s12889-020-09376-z)
Supplement: Supplementary file 1 — Additional file 1:. FTC old cohort 1975 wave 1 questionnaire English translation. Unofficial translation of mailed questionnaire used in wave 1 of the older Finnish Twin Cohort in 1975, originals in Finnish and Swedish. [file 12889_2020_9376_MOESM1_ESM.pdf]

TWIN RESEARCH, OLDER TWINS, QUESTIONNAIRE Q1975

1. Are you a twin? (you have/had a twin brother or twin sister)

1. no -> go to Q12
2. yes

2. My twin sibling (twin brother or sister)

1. lives in Finland
2. lives in Sweden
3. lives elsewhere abroad
4. died 19…..

3. Is your twin of the same sex as you?

1. no -> go to Q9
2. yes

4. My twin partner's Christian names are

____

Present surname is ____

Present address is____

5. Where you and your twin during childhood as alike as "two peas in the pod" or where you of ordinary family likeness?

1. "like two peas in the pod"
2. of ordinary family likeness
3. don't know

6. Where you and your twin so similar in appearance at school age that that people had difficulty in telling you apart?

1. no
2. yes
3. don't remember

7. Who could tell you apart?

	no	yes
your parents	1	2
your brothers or sisters	1	2
your classmates	1	2
other somewhat less familiar people	1	2

8. During your school years did you and your twin use any device to help people distinguish between you?

1. no
2. yes
3. don’t remember

9. How long did you live with your twin partner

1. I’m still living with him/her
2. We lived together until the age of___________years

10. How often do you meet or telephone your twin partner

1. daily or almost daily
2. about once a week
3. about once a month
4. about once every six month
5. less often
6. never

11**.** Which of you, you or your twin, was born first?

1. my twin partner
2. I
3. don’t know

12. Are you one of the triplets or quadruplets?

1. no
2. yes

13. When and where were you born?

____

date month year community of birth

14. Are you

1. single
2. married
3. re-married
4. living with someone but unmarried
5. divorced or separated
6. widow/widower

15. How tall are you

________________to the nearest centimeter

16. How much do you weight

________________to the nearest kilo

17. Are you losing weight because of obesity?

1. no
2. yes ->how?

	no	yes
by eating less and/or by changing a diet	1	2
with exercise	1	2
with anti-obesity drugs	1	2

18. Have you ever had any pain, discomfort, pressure or heaviness in your chest?

1. no-> go to Q24
2. yes-> go to next Q

19. Do you get it (pain, discomfort, pressure or heaviness) when you walk uphill or hurry on level ground?

1. no-> go to Q24
2. yes-> go to Q20
3. I never hurry or walk uphill-> do you get it when you walk at ordinary pace level ground

1. no -> go to Q24
2. yes -> go to next Q

20. What do you do if the pain, discomfort, pressure or heaviness comes while you are walking?

1. stop or slow down-> go to next Q
2. take a medicine and carry on -> go to next Q
3. carry on walking without taking any medicine-> go to Q24

21. If you stand still, what happens to it?

1. relieved-> go to next Q
2. not relieved-> go to Q24

22. How soon is it relieved?

1. less than 10 minutes-> go to next Q
2. more than 10 minutes –> go to Q24

23. Where do you feel it?	no	yes
middle upper part of the breastbone (A)	1	2
lower part of the breastbone (B)	1	2
left side on the chest (C)	1	2
in the left arm (D)	1	2
some other place (if so draw it in the picture)	1	2

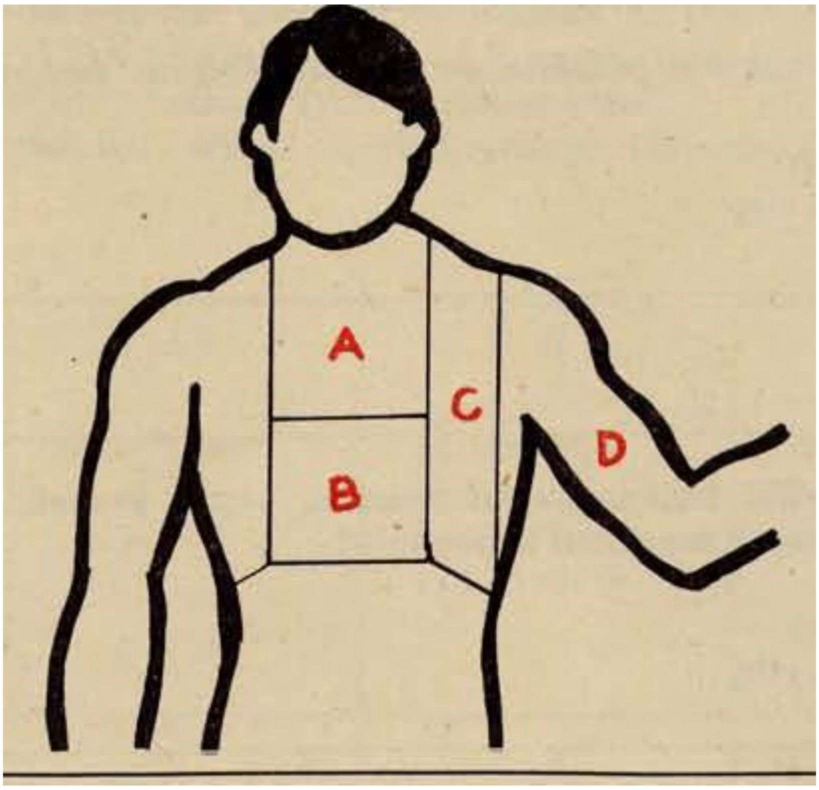

24. Do you regularly or for extended periods of time have a cough?

1. no->go to Q27
2. yes

25. How many months in a row do you cough per year?

1. less than three months in a row
2. more than three months in a row

26. For how many months in a row do you bring up phlegm from your chest per year?

1. less than three months in a row
2. more than three months in a row

27. Do you usually get short of breath when you walk uphill or climb stairs or hurry on level ground?

1. no
2. yes

28. Do you usually get short of breath when walking on level ground at an ordinary pace with people of your own age?

1. no
2. yes

29. Do you have to stop to breath because of the shortness of breath when you walk at your own pace on level ground (150 meters)?

1. no
2. yes

30. Do you usually get short of breath when standing still, for example when dressing or washing?

1. no
2. yes

31. Have you ever had severe pain across the front of your chest lasting a half hour or more?

1. no
2. yes

32. During the last years have you had pains in the back, shoulders or neck that make it difficult for you to work (circle also if not)?

	no	yes
pain in the back	1	2
pain in the shoulders	1	2
pain in the neck	1	2

33. Have you ever been told by the doctor that you have had? (circle also if you answer no)

	no	yes
chronic bronchitis……………………………………	1	2
emphysema…………………………………………….	1	2
asthma……………………………………………………	1	2
allergic cold e.g. hay fever………………………	1	2
allergic excema………………………………………	1	2
urticaria………………………………………………..	1	2
high blood pressure……………………………….	1	2
angina pectoris, in other words chest pain due to coronary disease……………………….	1	2
myocardial infarct "a coronary necrosis" in the breast muscle"……………………………….	1	2
stroke…………………………………………………….	1	2
peptic ulcers (stomach or duodenal ulcer)	1	2
gallstones……………………………………………….	1	2
diabetes………………………………………………….	1	2
gout………………………………………………………	1	2
operated or injected varicose veins…………	1	2
any other long term or serious illness, which?.....	1	2

…………………………………………………………………………………………

…………………………………………………………………………………………

34. This is a three-part question (A,B,C) , concerning your parents, brothers, sisters, children and spouse. Do first A, then B and finally C.

A. Record the year of birth of each of them (e.g. 1899, 1906) if you don't remember, please ask or estimate

B. Record the year of the death on the table for those who have died, for those who are alive please mark the line

C. If any of them have been hospitalized for or have died of myocardial infarct, record in the table the age at which the person got his/her first infarct. For those who have not had any, please mark the line.

	A. Year of birth	B. Year of the death	C. The age of first myocardial infarct
mother			
father			
spouse			
former spouse			
former spouse			
brother			
brother			
brother			
brother			
brother			
sister			
sister			
sister			
sister			
sister			
son			
son			
son			
son			
son			
daughter			
daughter			
daughter			
daughter			

35. As a child, I was

1. right handed
2. left-handed
3. both right-handed and left-handed

36. Do you write with your right hand today?

1. no
2. yes

37. If you were left-handed as a child, did they try to force you to write with your right hand?

1. no
2. yes

38. How many hours do you usually sleep at night?

1. less than 4
2. 5
3. 6
4. 7
5. 8
6. 9
7. 10 or more

39. Do you generally sleep well?

1. well
2. fairly well
3. fairly poorly
4. poorly
5. can’t say

40. Have you in your entire life smoked more than 5-10 packs of cigarettes?

1. no-> go to Q44
2. yes

41. Do you smoke or have you at some time smoked regularly, in the other words daily or almost daily?

1. no-> go to Q44
2. yes

42. How old were you when you began to smoke regularly?

…………………………..years

Do you smoke or did you smoke mainly

1. cigarettes with filter
2. cigarettes without filter
3. about equal amounts

43. Do you still smoke regularly?

1. No-> How old were you when you stopped smoking

………………………………years

How many cigarettes did you smoke on average per day before you stopped?

1. none
2. less than 5
3. 5-9
4. 10-14
5. 15-19
6. 20-24
7. 25-39
8. more than 40

2. Yes-> How many cigarettes do you smoke daily on average?

1. none
2. less than 5
3. 5-9
4. 10-14
5. 15-19
6. 20-24
7. 25-39
8. more than 40

44. Have you in your entire life smoked more than 50-75 cigars/cigarillos, or more than 3-5 packs of Pipe tobacco?

1. no -> go toQ53
2. yes

45. Do you smoke or have you at some time smoked cigars or cigarillos regularly i.e. daily or almost daily?

1. no -> go to Q49
2. yes

46. How old were you when you started to smoke cigars or cigarillos

……………………….years old

47. Have you stopped smoking cigars and cigarillos?

1. no
2. yes-> how old were you then ……………………………..years old

48. How many cigars and cigarillos do you smoke (alternatively) or did you smoked before you stopped?

   1. less than 3
   2. 3-9
   3. 10-19
   4. more than 20

49. Do you smoke or have you some time smoked pipe regularly i.e. daily or almost daily?

1. no-> go to Q53
2. yes

50. At what age did you start smoking the pipe?

…………………………………..years old

51. Have you stopped smoking the pipe?

1. no
2. yes-> At what age?.......................years old

52. How many packs of pipe tobacco per week do you smoke or (alternatively) did you smoke before stopping?

1. less than a half pack
2. half to a pack and a half
3. 2-3 packs
4. more than 4 packs

53. If you are or were a smoker, do you/did you inhale (draw smoke in to the lungs)?

1. no
2. yes

54. During the last year on how many days together have you used the followings types of the medicines (circle also even if you have not used any)?

	have no used	less than 10 days	10-59 days	60-180 days (2-6 months)	over 180 days (over 6 months)
Fortifying medicines (like iron or vitamin preparations)	1	2	3	4	5
Pain relievers	1	2	3	4	5
Antihypertensive drugs	1	2	3	4	5
Heart drugs	1	2	3	4	5
Antacids	1	2	3	4	5
Drugs for skin disorders	1	2	3	4	5
Sleeping pills	1	2	3	4	5
Tranquilizers	1	2	3	4	5

55. Do you now use contraceptive pills? (to be answered women only)

1. yes-> for how long have you used them ……………………………years
2. no-> have you used them earlier
   1. no
   2. yes-> for how long have you used them ……………………………years

56. How much of the following alcoholic beverages do you drink on average?

Beer
1. never
2. less than a bottle a week
3. 1-4 bottles a week
4. 5-12 bottles a week
5. 13-24 bottles a week
6. 25-47 bottles a week
7. more than 48 bottles a week

Wine or other mild alcohol beverages
1. never
2. less than a glass a week
3. a glass to 4 glasses a week
4. 1-2,5 bottles a week
5. 3-4,5 bottles a week

6. 5-9 bottles a week
7. more than 10 bottles a week

Hard liquor

1. never
2. less than a half bottle a month
3. a half bottle to a bottle and a half a month
4. 2- 3,5 bottles a month
5. 4-9 bottles a month
6. 10-19 bottles a month
7. more than 20 bottles a month

57. How often do you use alcohol? Which of the following alternatives best describes your use of beer, wine and hard liquor?

	never	on less than two days a month	on 3-8 days a month	on 9-16 days a month	over 16 days a month
Beer	1	2	3	4	5
Wine	1	2	3	4	5
Liquor	1	2	3	4	5

58. Does it happen that at least once a month and on the same occasion you drink more than five bottles of beer or more than bottle of wine or more than half a bottle of hard liquor?

1. no
2. yes

59. Has your alcohol consumption in the past been on average higher than it is today?

1. no-> go to Q61
2. yes

60. When your average alcohol consumption was at its highest, how much did you use?

Beer

8. never
9. less than a bottle a week
10. 1-4 bottles a week
11. 5-12 bottles a week
12. 13-24 bottles a week
13. 25-47 bottles a week
14. more than 48 bottles a week

Wine or other mild alcohol beverages

8. never
9. less than a glass a week
10. a glass to 4 glasses a week
11. 1-2,5 bottles a week
12. 3-4,5 bottles a week

13. 5-9 bottles a week
14. more than 10 bottles a week

Hard liquor

8. never
9. less than a half bottle a month
10. a half bottle to a bottle and a half a month
11. 2- 3,5 bottles a month
12. 4-9 bottles a month
13. 10-19 bottles a month
14. more than 20 bottles a month

61. How much do you usually drink the day of? (If you do not drink daily write 0)

Coffee……………………………cups

Tea…………………………………cups

How much sugar do you use in each cup?

0. none
1. one lump
2. two lumps
3. three lumps
4. four lumps or more

62. How much of your daily journey to work is spent in walking, cycling, running and/or cross-country skiing?

1. less than 15 min
2. 15 min or less than half an hour
3. half hour to less than hour
4. hour or more
5. I am presently not at work

63. Physical activity during leisure time (activity which does not occur at work or on the way to work). Here are five alternatives to choose from when deciding on the exercise you get during your leisure time. Which one applies best to you when considering the exercise you get during the year as a whole?

1. practically none
2. a little
3. a moderate amount
4. quite a lot
5. a great deal

64. Is your physical activity during leisure time about as tiring on average as:

1. walking
2. alternatively walking and jogging
3. jogging (light run)
4. running

65. Do you during your leisure time activity..

1. sweat profusely
2. sweat somewhat
3. not sweat at all

66. Does your leisure time activity generally cause breathlessness?

1. during almost the whole period of activity
2. in bouts during physical activity
3. only slightly
4. not at all

67. How long does the physical activity last at one session on average?

1. less than 15min
2. 15 minutes – less than half an hour
3. half an hour – less than an hour
4. an hour to less than two hours
5. over two hours

68. Presently how many times per month do you engage in physical activity during your leisure time?

1. less than once a month
2. 1-2 times a month
3. 3-5 times a month
4. 6-10 times a month
5. 11-19 times a month
6. more than 20 times a month

69. Have you ever over the age of 17 engaged in physical activity at least once a week that caused definite sweating and breathlessness?

1. no
2. yes-> how many years?
    1. less than half a year
    2. half a year- one year
    3. 2-4 years
    4. more than 5 years

70. How well do the following statements describe you? Choose the best alternative. The statements describes you very well, well, not very well, not at all

The statement describes me

	very well	well	not very well	not at all
In general I am unusually tense or nervous	1	2	3	4
There is a great deal of strain connected with my daily activities	1	2	3	4
At the end of the day I am completely exhausted mentally and physically	1	2	3	4
My daily activities are extremely trying and stressful	1	2	3	4

71. Do you have any pets?

1. no
2. yes-> which one.......................................

72. Do you have a dog?

1. no
2. yes-> What breed of dog.....................................

73. What kind of education have you had, and what courses have you taken?

1. less than primary school
2. primary school or its equivalent
3. primary school or its equivalent and at least one year vocational training
4. junior high school
5. junior high school and at least one year vocational training (or senior high school)
6. high school graduate
7. high school graduate and at least one year vocational training (or university studies)
8. university degree or equivalent
9. other education, what?...

74. Are you at present

1. working
2. a house wife
3. an old age pensioner
4. on disability or illness pension

5. a student, scholar
6. unemployed, looking for work
7. something else, what?..

75. At the present moment, are you

1. working for somebody else on a monthly or hourly salary basis
2. working for somebody on contractual basis
3. self employed (non farm)
4. farmer
5. I am not working at the present moment
6. I have never worked

76. What kind of work do you or have you done (If not now working)? Describe your work as accurately as possible…….

…….

77. How much is your monthly income today (without deducting taxes)?

1. none
2. less than 600mk
3. 601-1200mk
4. 1201-1800mk
5. 1801-2400mk
6. 2401-3000mk
7. 3000-3600mk
8. more than 3600mk

78. Is your present work, or the work which you last did, in your opinion

1. very monotonous
2. quite monotonous
3. quite varying
4. very varying
5. I can't say
6. I have never worked

79. Is your present work or the work which you last did

1. mainly indoors
2. mainly outdoors
3. equally both
4. I have never worked

80. Is your present work or the work which you last did

1. mainly day work
2. mainly night work
3. mainly shift work
4. I have never worked

81. What kind of work did/do you do? The present work or the work you last did

1. mainly sedentary work, which requires very little physical activity
2. work which involves standing and walking, but no other physical activity
3. work which in addition to standing and walking requires lifting and carrying
4. heavy physical work

82. Are you the primary provider of your family?

1. yes-> go to Q84
2. no-> What is the profession of the primary provider of your family? (describe as accurately as possible)……

83. What is the monthly income of the primary provider of your family? (without deducting taxes)

1. less than 600mk
2. 601-1200mk
3. 1201-1800mk
4. 1801-2400mk
5. 2401-3000mk
6. 3000-3600mk
7. more than 3600mk

84. In the following we will present some questions that deal with the way people feel and act. For each question circle that alternative (no or yes) that best describes the way you generally feel and act. Circle the alternative which first comes into your mind. *no-1 yes-2*

	No	yes
Do you like to have lots of things going on around you?	1	2
Are you often uneasy, feeling that there is something you want without knowing what it is?	1	2
Do you almost always have an answer ready when spoken to?	1	2
Are you sometimes happy and sometimes sad without any special reason?	1	2
Do you prefer to keep to the background in the company of people?	1	2
Do you regard yourself as happy and carefree?	1	2
Do you often reach decisions too late?	1	2
Do you often feel tired and listless without any special reason?	1	2
Do you have a lively manner / Are you lively and talkative?	1	2
Can you describe your thoughts in words quickly?	1	2

Are you often lost in your thoughts (even if others expect you to participate in conversation)?	1	2
Do you have anything against selling lottery tickets or asking people for money for some purpose you value yourself?	1	2
Are you extremely sensitive in some respects?	1	2
Do you sometimes feel so restless that you cannot sit still?	1	2
Do you have difficulties in falling asleep in the evenings?	1	2
Do you usually keep your distance with others except your friends?	1	2
Do you have any nervous problems?	1	2
Do you like to crack jokes and tell funny stories to your friends?	1	2
Do you think you usually worry too long after a distressing situation?	1	2

85. Do you feel that your life at the present moment is very interesting, fairly interesting, fairly boring or very boring?

1. very interesting
2. fairly interesting
3. fairly boring
4. very boring
5. don't know

86. Do you feel that at the present moment your life is very happy, fairly happy, fairly sad or very sad very happy?

1. very happy
2. fairly happy
3. fairly sad
4. very sad
5. don't know

87. Do you feel that at the present moment your life is very easy, fairly easy, fairly hard or very hard?

1. very easy
2. fairly easy
3. fairly hard
4. very hard
5. don't know

88. Do you feel that at the present moment you are very lonely, fairly lonely or not at all lonely?

1. very lonely
2. fairy lonely

3. not at all lonely
4. don’t know

89. Here is 10 traits and characteristics. Words are opposites and you should circle the number that best describes what kind of the person you are. t.ex fast---slow, nr 1 means fast, 2 quite fast…4 quite slow and 5 slow.

never late	1	2	3	4	5	casual about appointments
not competitive	1	2	3	4	5	very competitive
sensitive	1	2	3	4	5	brave
rushed	1	2	3	4	5	not rushed
takes thing one at time	1	2	3	4	5	tries to do many thing at time
lazy	1	2	3	4	5	diligent
fast	1	2	3	4	5	slow
“sits” on feelings	1	2	3	4	5	expresses feelings
many interests	1	2	3	4	5	few interests
short-tempered	1	2	3	4	5	peaceful

90. How many times during your life have you change your residence?

0. none
1. once
2. twice
3. 3-4
5. 5-8
9. 9-16
17. more than 17

91. What were the reasons for the changing of residence?

	not once	once	2-3 times	4-7 times	more than 7 times
chance of one’s own or family members job	0	1	2	4	7
due to studying or going to school	0	1	2	4	7
due to lack of jobs	0	1	2	4	7
because of buying your own home	0	1	2	4	7
due to termination of rental agreement	0	1	2	4	7
due to one’s own or parent’s divorce	0	1	2	4	7

I was dissatisfied with the relationship	0	1	2	4	7
due to illness of one's own or family members	0	1	2	4	7
due to war	0	1	2	4	7
due to other reason	0	1	2	4	7

92. How many times during your life have you change your job?

0. I have never worked->go to Q94
0. never-> go to Q94
1. once
2. twice
3. 3-4 times
5. 5-8 times
9. 9-16 times
17. 17-39 times
40. more than 40 times

93. What were the reasons for the changing the job?

	**not once**	once	2-3 times	4-7 times	more than 7 times
I changed my place of residence	0	1	2	4	7
I got more interesting job	0	1	2	4	7
I got better salary	0	1	2	4	7
due to studying	0	1	2	4	7
due to termination of the employment contract	0	1	2	4	7
due to illness	0	1	2	4	7
I was dissatisfied with the relationship at work	0	1	2	4	7
the job was too heavy	0	1	2	4	7
due to other reason	0	1	2	4	7

94. How many times in your life have you been unemployed?

0. never->go to Q94
0. I have never worked->go to Q94
1. once
2. twice

3. 3-4 times
5. 5-8 times
9. more than 9 times

95. What were the reasons for your unemployment?

	not once	once	twice	3-4 times	more than 5 times
the contract ended due to illness	0	1	2	3	5
the contract ended due to accident	0	1	2	3	5
the contract ended due to other reason	0	1	2	3	5

96. If your permanent address has changed write your new address here below

……
……

97. Do you have any comments or objections to this questionnaire?

1. no
2. yes-> what?
   ……
   ……
   ……
