# Supplementary material for: Changes in self-reported sleep duration with age - a 36-year longitudinal study of Finnish adults
Source: BMC Public Health. 2020 Sep 9;20:1373. doi: 10.1186/s12889-020-09376-z (PMC7487757; doi:10.1186/s12889-020-09376-z)
Supplement: Supplementary file 2 — Additional file 2:. FTC old cohort 1981 wave 1 questionnaire English translation. Unofficial translation of mailed questionnaire used in wave 2 of the older Finnish Twin Cohort in 1981, originals in Finnish and Swedish. [file 12889_2020_9376_MOESM2_ESM.pdf]

1 My twin sibling (= twin brother or sister)

1 lives in Finland
2 lives in Sweden
3 lives elsewhere abroad
4 died 19..

2 My twin partner's Christian names are

....

present surname is ....

present address is ....

....

3 Were you and your twin partner during childhood as alike as "two peas in a pod" or were you of ordinary family likeness?

1 "like two peas in a pod"
2 of ordinary family likeness
3 don't know

4 Were you and your twin partner so similar in appearance at school age that people had difficulty in telling you apart?

1 no
2 yes
3 don't remember

5 How long did you live with your twin partner?

1 I am still living with him/her
2 we lived together until the age of ..... years.

6 How often do you meet or telephone your twin partner

1 daily or almost daily
2 about once a week
3 about once a month
4 about once every six month
5 less often
6 never

7 When and where were you born?

....
day month year community of birth

8 Are you

1 single
2 married
3 re-married
4 living with somebody, but unmarried
5 divorced or separated
6 widow/widower

9 How tall are you

.... to the nearest centimeter.

10 How much do you weigh

.... to the nearest kilo

11 Have you ever had any pain, discomfort, pressure or heaviness in your chest?

1 no ------> go to Q 17
2 yes -----> go to next Q

12 Do you get it (pain, discomfort, pressure or heaviness) when you walk uphill or hurry on level ground?

1 no ------> go to Q 17
2 yes -----> go to Q 13
3 I never hurry or walk uphill ---->
  Do you get it when walk at ordinary pace on level ground?
    1 no ------> go to Q 17
    2 yes -----> go to next Q

13 What do you do if the pain, discomfort, pressure or heaviness comes while you are walking?

1 stop or slow down -----> go to next Q
2 take a medicine and carry on ---> go to next Q
3 carry on walking without taking any medicine
  ----> go to Q 17

14 If you stand still, what happens to it?

1 relieved -----> go to next Q
2 not relieved -----> go to Q 17

15 How soon is it relieved?

1 less than 10 minutes -----> go to next Q
2 more than ten minutes -----> go to Q 17

16 Where do you feel it?

	no	yes
middle upper part of the breastbone (A)	1	2
lower part of the breastbone (B)	1	2
left side of the chest (C)	1	2
in the left arm (D)	1	2
some other place (if so, draw it in the picture)	1	2

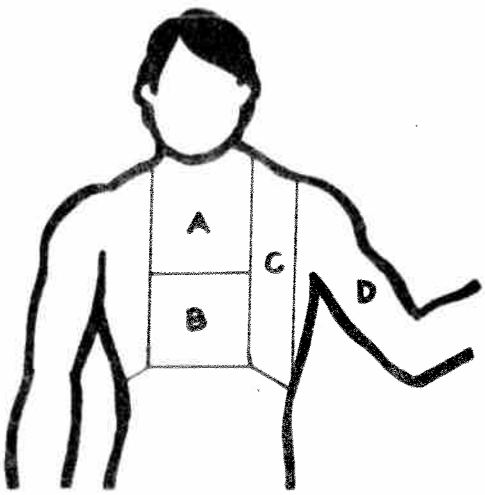

17 Do you regularly or for extended periods of time have a cough?

1 no -----> go to Q 20
2 yes

18 How many months in a row do you cough per year?

1 less than three months in a row
2 more than three months in a row

19 For how many months in a row do you bring up phlegm from your chest per year?

1 less than three months in a row
2 over than three months in a row

20 Do you usually get short of breath when you walk uphill, climb stairs or hurry on level ground?

1 no
2 yes

21 Do you usually get short of breath when walking on level ground at an ordinary pace with people of your own age?

1 no
2 yes

22 Do you have to stop to breathe because of shortness of breath when you walk at your own pace on level ground (150 metres)?

1 no
2 yes

23 Do you usually get short of breath when standing still, for example when dressing or washing?

1 no
2 yes

24 Have you ever had severe pain across the front of your chest lasting a half hour or more?

1 no
2 yes

25 a) Do you get pain in the legs on walking?

1 no ----> go to Question 26
2 yes

b) Does this pain ever begin when you are standing still or sitting?
1 no
2 yes ---->go to question 26

c) In what part of your legs do you feel it?

1 in both calves
2 in one calf
3 in neither calf ----> go to question 26

d) Do you get the pain if you walk uphill or hurry?
1 no ------>go to question 26.
2 yes ---> continue from part e
3 I never walk uphill or hurry --->
Does the pain come if you walk at an slow pace on the level?
1 no ------>go to question 26.
2 yes

e) Does the pain ever disappear while you are walking?
1 no
2 yes

f) What do you do if you get it when you are walking
1 stop or slow down
2 carry on

g) What happens to it if you stand still?

1 relieved
2 not relieved

h) How soon?

1 less than 10 minutes
2 more than 10 minutes

26 Have you had any of the following symptoms during last month?

	Daily or almost daily	About once a week	Less often	Not at all
Heart palpitations without any physical effort...........	1	2	3	4
Irregular heart beats.........	1	2	3	4
Chest pain while angry or emotionally upset.............	1	2	3	4
Perspiration without physical effort........................	1	2	3	4
Facial blushing..............	1	2	3	4

27 Has a nurse, public health nurse or doctor measured your blood pressure during the past five years?

1 No ---> got to the next question
2 I don't remember ---> got to the next question
3 Yes and the blood pressure was
    1 normal
    2 slightly elevated and no medicine was prescribed
    3 elevated, and medicine prescribed that has now been stopped
    4 permanantly elevated, and a continuous medication was prescribed.

Where was it last measured?

____

28 Has the fat content (cholesterol) of your blood been determined during the past five years?

1 no
2 I don't know
3 yes, and the cholesterol level was
    1 below normal
    2 normal
    3 elevated

Where was it last measured?

____

29 During the last years have you had pains in the back, shoulders or neck that make it difficult for you to work (circle also if not)?

	no	yes
pain in the back	1	2
pain in the shoulders	1	2
pain in the neck	1	2

30 Have you ever been told by a doctor that you have had? (circle also if you answer no)

	no	yes
chronic bronchitis......................	1	2
emphysema...............................	1	2
astma...................................	1	2
allergic cold e.g. hay fever............	1	2
allergc excema..........................	1	2
epilepsy (or 'seizures')................	1	2
high blood pressure.....................	1	2
angina pectoris, in other words chest pain due to coronary disease............	1	2
myocardial infarct, "a coronary", necrosis in the heart muscle............	1	2
peptic ulcers (stomach or duodenal ulcer).................................	1	2
migraine	1	2
rheumatoid artritis.....................	1	2
joint degeneration or athrosis..........	1	2
gallstones..............................	1	2
diabetes................................	1	2
Parkinson's disease	1	2
psoriasis	1	2
any other long or serious illness, which?	1	2
....		

31 Do you have tremors of the hands or feet?

1 no

2 yes ---> Is this tremor associated with clumsiness or stiffness of the hands or feet?
1 yes
2 no

32 Have you at some time in your life had, or do you at present have ?

	yes	no
Recurrent dizzy spells.................	1	2
Black outs.............................	1	2
Convulsive fits........................	1	2
Other consciousness disturbances	1	2

33 Have you had or do you presently have a continuous medication for the disturbances mentioned in questio 32?

1 no
2 yes

34 As a child did you had febrile convulsions?

1 no
2 yes
3 don't know

35 Do you have headaches?

1 daily or almost daily
2 several times a week
3 about once weekly
4 about once a month
5 several times a year (but not every month)
6 once a year or less often
7 I have had practically speaking no headaches

36 Do you have headache attacks?

1 no ---> go to Q 37
2 yes --> answer the following questions:

	yes	no
Vision disturbances during the attacks	1	2
Nausea, retching or vomiting during the headache	1	2
Unilateral headache................	1	2

37 How many hours do you usually sleep at night?

1 6 hours or under 6 hours
2 6.5 hours
3 7 hours
4 7.5 hours
5 8 hours
6 8.5 hours
7 9 hours
8 9.5 hours
9 10 hours or more

38 How many hours of sleep per night do you need in order to be in good working condition the next day ?

1 6 hours or under 6 hours
2 6.5 hours
3 7 hours
4 7.5 hours
5 8 hours
6 8.5 hours
7 9 hours
8 9.5 hours
9 10 hours or more

39 Do you generally sleep well?

1 well
2 fairly well
3 fairly poorly
4 poorly
5 can't say

40 Do you snore in your sleep?

1 never
2 sometimes
3 often
4 almost always
5 cannot say

41 Have you in your entire life smoked more than 5-10 packs of CIGARETTES?

1 no -----> go to Q 45
2 yes

42 Do you smoke or have you at some time smoked regularly, in other words daily or almost daily?

1 no ----> go to Q 45
2 yes

43 How old were you when you began to smoke regularly?

..... years old

Do you smoke or did you smoke mainly

1 cigarettes with filters
2 cigarettes without filters
3 about equal amounts

Do you smoke or did you smoke mainly

1 factory made cigarettes
2 self-rolled cigarettes
3 about equal amounts

44 Do you still smoke regularly?

1 no ---> How old were you when you stopped smoking?
..... years old
How many did you smoke on average per day before you stopped?
1 none
2 less than 5 cigarettes
3 5- 9 cigarettes
4 10-14 cigarettes
5 15-19 cigarettes
6 20-24 cigarettes
7 25-39 cigarettes
8 over 40 cigarettes

2 yes ---> How many cigarettes do you smoke daily on average?
1 none
2 less than 5 cigarettes
3 5- 9 cigarettes
4 10-14 cigarettes
5 15-19 cigarettes
6 20-24 cigarettes
7 25-39 cigarettes
8 over 40 cigarettes

Next will be asked the tar, carbon monoxide and nicotine content of the cigarettes you presently smoke (The contents are marked on the side of your cigarette box).

What is the tar content of the brand you smoke?

1 9 mg or less
2 10-14 mg
3 15-17 mg
4 18 mg or more

What is the carbon monoxide content of the brand you smoke?

1 8 mg or less
2 9-12 mg
3 13-15 mg
4 16 mg or more

What is the nicotine content of the brand you smoke?

1 0.5 mg or less
2 0.6-0.8 mg
3 0.9-1.1 mg
4 1.2 mg or more

45 Have you in your entire life smoked more than 50-75 CIGARS/CIGARILLOS, or more than 3-5 packs of PIPE TOBACCO?

1 no -----> go to Q 54
2 yes

46 Do you smoke or have you at some time smoked cigars or cigarillos regularly i.e. daily or almost daily?

1 no -----> go to Q 50
2 yes

47 How old were you when you started smoking cigars or cigarillos?

..... years old

48 Have you stopped smoking cigars and cigarillos?

1 no
2 yes -----> How old were you then? ....years old.

49 How many cigars and cigarillos do you smoke (alternatively) or did you smoke before you stopped?

1 less than 3 per day
2 3-9 per day
3 10-19 per day
4 over 20 per day

50 Do you smoke or have you at some time smoked the pip regularly, i.e. daily or almost daily?

1 no -----> go to Q 54
2 yes

51 At what age did you start smoking the pipe?

..... years old.

52 Have you stopped smoking the pipe?

1 no
2 yes -----> At what age? ..... years old.

53 How many packs of pipe tobacco per week do you smoke or (alternatively) did you smoke before stopping?

1 less than a half pack
2 half to a pack and a half
3 2-3 packs
4 more than 4 packs

54 If you are or were a smoker, do you/did you inhale (draw smoke into the lungs)?

1 no
2 yes

55 During the last year on how many days together have you used the followings types of medicines (circle also, even if you have not used any)?

	have not used	less than 10 days	10-59 days	60-180 days (2-6 months)	over 180 days (over 6 months)
Fortifying medicines (like iron or vitamin preparations).........	1	2	3	4	5
Pain relievers........	1	2	3	4	5
Antihypertensive drugs.................	1	2	3	4	5
Heart drugs...........	1	2	3	4	5
Antacids..............	1	2	3	4	5
Drugs for skin disorders.............	1	2	3	4	5
Sleeping pills.........	1	2	3	4	5
Tranquillizers.........	1	2	3	4	5

56 Do you now use contraceptive pills? (to be answered women only)

1 yes ----> For how long have you used them?

..... years

2 no ----> Have you used them earlier?

1 no
1 yes ----> for how long have you used them? ..... years

When you were or are on the pill, what brand did you mainly use ?

57 How much of the following alcoholic beverages do you drink on average?

Beer
1 never
2 less than a bottle a week
3 1-4 bottles a week
4 5-12 bottles a week
5 13-24 bottles a week
6 25-47 bottles a week
7 more than 48 bottles a week

Wine or other mild alcoholic beverages
1 never
2 less than a glass a week
3 a glass to 4 glasses a week
4 1-2,5 bottles a week
5 3-4,5 bottles a week
6 5-9 bottles a week
7 more than 10 bottles a week

Hard liquor
1 never
2 less than a half bottle per month
3 a half bottle to a bottle and a half per month
4 2-3,5 bottles a month
5 4-9 bottles a month
6 10-19 bottles a month
7 more than 20 bottles a month

58 How often do you use alcohol? Which of the following alternatives best describes your use of beer, wine and hard liquor?

	Never	On less than two days a month	On 3-8 days a month	On 9-16 days a month	Over 16 days a month
Beer........	1	2	3	4	5
Wine........	1	2	3	4	5
Liquor......	1	2	3	4	5

59 Does it happen that at least once a month and on the same occasion you drink more than five bottles of beer or more than bottle of wine or more than half a bottle of hard liquor?

1 no
2 yes

60 How often have you passed out while using alcohol during the last year ?

1 not once
2 once
3 two-three times
4 four-six times
7 seven times or more

61 How much do you usually drink a day of? (if you do not drink daily, write 0)

COFFEE ..... cups

TEA ..... cups

How much sugar do you use in each cup?

0 none
1 one lump
2 two lumps
3 three lumps
4 four lumps or more

62 How many glass of milk do you drink a day?

0 none
1 1-2 glasses
3 3-5 glasses
6 6 glasses or more

63 How many glassess of water do you drink daily ?

0 none
1 1-2 glasses
3 3-5 glasses
6 6 glasses or more

What water do you mainly drink?
1 tapwater
2 water from my own well
3 bottled or bagged water
4 I don't drink water

64 How many eggs do you eat daily?

0 none
1 one
2 two
3 three or more

65 On how many meals or snacks do you eat daily vegetables or fruit (at least the equivalent of one tomato)?

0 not one
1 1-2 meals or snacks
3 3-5 meals or snacks
6 6 or more meals or snacks

66 Do you like salty meals?

1 quite a lot
2 somewhat
3 not at all

67 Do you eat at least daily 2-3 mouthfuls of salty food (saltfish, salt cucumber etc)?

1 no
2 yes

68 What fat do you spread on your bread?

1 none
2 mostly margerine
3 mostly butter
4 about equal amount of both
5 I hardly eat bread

69 How much of your daily journey to work is spent in walking, cycling, running and/or cross-country skiing?

1 less than 15 min.
2 15 min - less than half an hour
3 half hour to less than an hour
4 hour or more
5 I am presently not at work

70 Physical activity during leisure time (activity which does not occur at work or on the way to work). Here are five alternatives to choose from when deciding on the exercise you get during your leisure time. Which one applies best to you when considering the exercise you get during the year as a whole?

1 practically none
2 a little
3 a moderate amount
4 quite a lot
5 a great deal

71 Is your physical activity during leisure time about as tiring on average as:

1 walking
2 alternatively walking and jogging
3 jogging (light run)
4 running

72 Do you during your physical activity in leisure time generally?

1 sweat profusely
2 sweat somewhat
3 not sweat at all

73 Does your leisure time physical activity generally cause breathlessness

1 during almost the whole period of activity
2 in bouts during the physical activity
3 only slightly
4 enot at all

74 How long does the physical activity last at one session on average?

1 less than 15 min.
2 15 min. but under 1/2 hour
3 1/2 hour, but under 1 hour
4 1 hour to under 2 hours
5 over two hours

75 Presently how many times per month do you engage in physical activity during your leisure time?

1 less than once a month
2 1-2 times a month
3 3-5 times a month
4 6-10 times a month
5 11-19 times a month
6 more than 20 times a month

76 Have you during the last five years engaged in physical activity at least once a week that caused definite sweating and breathlessness?

1 no
2 yes ----> For how many years?
1 less than six months
2 half a year to a year
3 2-4 years
4 over 5 years

77 How well do the following statements describe you? Choose the best alternative. The statement describes you very well, well, not very well and not at all.

	The statement describes me:			
	very well	well	not very well	not at all
In general I am unusually tense or nervous.........	1	2	3	4
There is a great deal of strain connected with my daily activities.........	1	2	3	4
At the end of the day I am completely exhausted mentally and physically..	1	2	3	4
My daily activities are extremely trying and stressful................	1	2	3	4

78 What kind of an education have you had, and what courses have you fillowed?

1 less than primary school (6 years)
2 primary school or its equivalent (6-7 years)
3 primary school or its equivalent and at least one year of vocational training
4 junior high school (9 years)
5 junior high school and at least one year of vocational training or senior high school
6 high school graduate (12-13 years)
7 high school graduate and at least one year of vocational trainig or university
8 university degree or equivalent
9 other education, what?

....

79 Are you at present

1 working
2 a housewife
3 an old age pensioner
4 on diability or illness pension
5 a student, scholar
6 unemployed, looking for work
7 something else, what?

....

80 At the present moment are you

1 working for somebody else on a monthly or hourly salary basis
2 working for somebody on a contractual basis
3 self-employed (non-farm)
4 farmer
5 I am not working at the present moment
6 I have never worked

81 How much overtime or extra work have you done (=work in addition to a normal work day) on average during the past year?

1 none or less than an hour a day
2 on average one hour a day
3 on average 2-3 hours a day
4 on average 4 hours or more a day
5 I an not working

82 What kind or work do you or have you done (if not now working)? Describe your work as accurately as possible.

....

....

83 During your whole life have you been in contact at work or during leisure activities by breathing or touching daily or almost daily with chemical liquids, vapors or gases? (Eg: acids, bases, poisons, paints, glues, pesticides, etc)

1 never ---->go to question 84
2 less than one month together
3 one to six months together
4 half a year to one year together
5 two to four years together
6 five years or longer together
7 cannot say

Describe in detail in which work or leisure activity and with which chemicals you have been principally in contact (had to breathe or touch)

....
....
....
....
....

84 Is your present work, or the work which you last did, in your opinion?

1 very monotonous
2 fairly monotonous
3 fairly varying
4 very varying
5 can't say
6 I have never been at work

85 How much can you determine the pace of work in your present work ?

1 I can determine the pace quite freely
2 I can influence it somewhat
3 I am doing work at a forced or nearly forced pace
4 I am not working

86 Is your present work, or the work which you last did

1 mainly indoors
2 mainly outdoors
3 equally both
4 I have never worked

87 Is your present work, ot the work which you last did

1 mainly daywork
2 mainly nightwork
3 mainly shiftwork
4 I have never worked

88 What kind of work did/do you do? The present work or the work which you last did?

1 mainly sedentary work, which requires very little physical activity
2 work which involves standing and walking, but no other physical activity
3 work which in addition to standing and walking requires lifting and carrying
4 heavy physical work

89 On working day mornings I am in general

1 very vigorous
2 fairly vigorous and rested
3 fairly tired
4 very tired

90 How long does it take you to 'get going' in the morning when you wake up

1 10 minutes or less
2 more than 10 minutes but under 20 minutes
2 more than 20 minutes but under 40 minutes
4 over 40 minutes

91 Try to say to what extent you are a 'morning person or an evening person '.

1 I an clearly a morning person (bright in the morning and sleep in the evening
2 I am somewhat a morning person
3 I am somewhat an evening person (sleepy in the morning and bright in the evening)
4 I am clearly an evening person

92 In the following we will present some questions that deal with the way people feel and act. For each question circle that alternative (yes or no) that best describes the way you generally feel and act. Don't think for a long time, rather circle that alternative which first comes into your mind.

	No	Yes
Do you like to have lots of things going on around you?	1	2
Are you often uneasy, feeling that there is something you want without knowing it?	1	2
Do you almost always have an answer ready when spoken to?	1	2
Are you sometimes happy or sometimes sad without any special reason?	1	2
Do you prefer to keep to the background in the company of people?	1	2
Do you regard yourself as happy and carefree?	1	2
Do you often reach decisions too late?	1	2
Do you often feel tired and listless without any special reason?	1	2
Do you have a lively manner?	1	2
Can you quickly describe your thoughts in words?	1	2
Are you often lost in your thoughts?	1	2
Do you have anything against selling things or asking people for money for some charitable purpose?	1	2
Are you extremely sensitive in any respects?	1	2
Are you ever too restless to sit still?	1	2
Do you have difficulties in falling asleep?	1	2
Do you keep things to yourself except with good friends?	1	2
Do you have any nervous problems?	1	2
Do you like to crack jokes and tell funny stories to your friends?	1	2
Do you usually worry a long time after a distressing incident?	1	2

People use different words and phrases to describe their own life. We want you to answer the following question in the way you feel.

93 Do you feel that your life at the present moment is wery interesting, fairly interesting, fairly foring or very boring?

1 very interesting
2 fairly interesting
3 fairly boring
4 very boring
5 don't know

94 Do you feel that at the present moment your life is very happy, fairly happy, fairly sad or very sad?

1 very happy
2 fairly happy
3 fairly sad
4 very sad
5 don't know

95 Do you feel that at the present moment your life is very eary, fairly easy, fairly hard or very hard?

1 very easy
2 fairly easy
3 farly hard
4 very hard
5 don't know

96 Do you feel that at the present moment you are very lonely, fairly lonely or not at all lonely?

1 very lonely
2 fairly lonely
3 not at all lonely
4 don't know

97 In the following a number of events that can happen in life are listed. Have any of these happened to you?

	The event happened to me:			
	Not at all	During the last six months	During the last five years	Earlier
Death of spouse............	1	2	3	4
Death of close relative or good friend................	1	2	3	4
Miscarriage in the family or in a close person........	1	2	3	4
Marked change in the health of a family member (not death).....................	1	2	3	4
Difficulties of a sexual nature......................	1	2	3	4
Marked difficulties with superiors, colleagues or subordinates at work........	1	2	3	4
Marked worsening in financial situation.........	1	2	3	4
Increase in family size (eg birth of child, adoption of a child, relative come to live in the family)......	1	2	3	4
Change of home.............	1	2	3	4
Departure of family member from home..................	1	2	3	4
Divorce or separation.......	1	2	3	4
Break in long-term human relationship (not divorce)..	1	2	3	4
Loss of job................	1	2	3	4
Change to another kind of work.......................	1	2	3	4
Marked increase in difficulties with spouse (not divorce)..............	1	2	3	4
Marked increase of res-posibility at work..........	1	2	3	4
Marked increase in work load	1	2	3	4
Bank loan equivalent to over six month's earninigs.......	1	2	3	4
Disease or injury causing over three weeks work disabilty..................	1	2	3	4
Living apart from spouse because of work............	1	2	3	4
Marked change for the better in life.............	1	2	3	4

98 Every human being has a certain idea about himself. We ask you to describe with the words presented, what kind of a person you think you are. The words are opposites and represent traits and characteristics as far away from each other as possible.

In between the words you can find five lines. Circle one of the lines between the words, nearer by the word you think better describes you.

Examples:

Cheerful - - - - - sad
This way you will show to be sad.
Blonde - - - - - dark
This way you will show to be more blonde than dark.

Plesase, answer openly, how you really would describe yourself, not by the opinions you think other people have, or how you would perhaps wish to be.

Work fast, do not get too much stuck with any details. Use even the strongest expressions courageously and mark the middle circle only in case both of the characteristics really seem to describe you evenly well or poorly.

Never late	- - - - -	Casual about appointments
Not competitive	- - - - -	Very competitive
Open	- - - - -	Reserved
Rushed	- - - - -	Not rushed
Takes thing one at a time	- - - - -	Tries to do many thing at a time
Rarely quarrelsome	- - - - -	Quarrelsome
Fast	- - - - -	Slow
"Sits" on feelings	- - - - -	Expresses feelings
Many interests	- - - - -	Few interests
Doe not get angry easily	- - - - -	Gets angry easily
Determined	- - - - -	Uncertain
Communicative	- - - - -	Uncommunicative
Submissive	- - - - -	Dominating
Self-confident	- - - - -	Not sure of oneself
Always honest	- - - - -	Sometimes dishones in little things
Quiet	- - - - -	Talkative
Diligent	- - - - -	Lazy
Gets easily irritated	- - - - -	Doesn't get irritated
Doesn't always tell the whole truth	- - - - -	Tells always the whole truth

In the following questions we ask you to describe your relationship to other family members in **childhood**.

Distant relationship to father	- - - - -	Close relationship to father
Distant relationship to mother	- - - - -	Close relationship to mother
Distant relationship to twin brother/ sister	- - - - -	Close relationship to twin brother/ sister

99 If your permanent address has changed, write in your new address below

....

....

100 Do you have any comments or objections to this questionnaire?

1 no
2 yes ----> What?

....

....

WE ASK YOU TO CHECK THAT YOU ANSWERED ALL THE QUESTIONS CORRECTLY, PLEASE.

THANK YOU FOR YOUR CO-OPERATION
