# Supplementary material for: Changes in self-reported sleep duration with age - a 36-year longitudinal study of Finnish adults
Source: BMC Public Health. 2020 Sep 9;20:1373. doi: 10.1186/s12889-020-09376-z (PMC7487757; doi:10.1186/s12889-020-09376-z)
Supplement: Supplementary file 3 — Additional file 3:. FTC old cohort 1990 wave 3 questionnaire English translation. Unofficial translation of mailed questionnaire used in wave 3 of the older Finnish Twin Cohort in 1990, originals in Finnish and Swedish. [file 12889_2020_9376_MOESM3_ESM.pdf]

TWIN RESEARCH, OLDER TWINS, QUESTIONNAIRE Q1991

1. How long did you live with your co-twin?

    1. I am still living with him/her

    2. We have lived together until the age of ___ years.

2. How much of your free time did you spend with your co-twin at the age of 15-16?

    1. nearly all of it
    2. I spent somewhat more of my free time with my twin than with other people
    3. I spent somewhat more of my free time with others than with my twin.
    4. I spent most of my free time with others than my twin
    5. we lived apart and don't have the opportunity to spend time together
    6. I cannot say

3. How often do you meet or communicate to your co-twin nowadays?

    1. daily
    2. about once a week
    3. about once a month
    4. about once every six month
    5. less often/less frequently
    6. never

4. Are you

    1. single
    2. married
    3. re-married
    4. living with somebody, but unmarried
    5. divorced or separated
    6. widow/widower

5. Do you have children aged 0-6 in your household at this time?

    1. no
    2. yes, how many?..

6. Do you have school children (7-16) in your household at this time?

    1. no
    2. yes, how many?...

7. Has a nurse, public health nurse or doctor measured your blood pressure during the past five years?

1. no
2. I don't remember
3. yes-> and the blood pressure was?
1. normal
2. slightly elevated and no medicine was prescribed
3. elevated, and medicine prescribed that has now been stopped
4. permanently elevated, and a continuous medication was prescribed

8. Has the fat content (cholesterol or fp-chol) of your blood been determined the past five years?

1. no
2. I don't know
3. yes-> and the cholesterol was
1. below normal,
2. normal
3. elevated
4. I don't know the value

What was the cholesterol value of the last measurement?

1. below 4
2. 4-4,9
3. 5-5,9
4. 6-6,9
5. 7-7,9
6. 8-8,9
7. ≥9

9. When was the last time you visited eye doctor?

1. less than 1 year ago
2. 1-3 years ago
3. 3-6 years ago
4. more than 6 years ago
5. never visited
6. don't remember

10. Has your doctor prescribed you eyeglasses or contact lenses?

	yes	no
eyeglasses	1	2
contact lenses	1	2

11. If you have eyeglasses or contact lenses, are they
1. distance spectacles
2. reading glasses
3. both distance spectacles and reading glasses or bifocals

12. Do you regularly or for extended periods of time have a cough?

1. no -> go to Q15
2. yes

13. How many months in a row do you cough per year?

1. less than three months in a row
2. more than three months in a row

14. For how many months in a row do you bring up phlegm from your chest per year?

1. less than three months in a row
2. more than three months in a row

15. During the last year have you had sore throat?

1. no
2. 1-2 times
3. 3-5 times
4. more than 5 times

16. Do you usually get short of breath when you walk uphill, climb stairs or hurry on level grounds?

1. no
2. yes

17. Do you usually get short of breath when walking on level ground at an ordinary pace with people of your own age?

1. no
2. yes

18. Do you have to stop to breathe because of shortness of breath when you walk at your own pace on level ground 150 meters?

1. no
2. yes

19. Do you usually get short of breath when standing still, for example when dressing or washing?

1. no
2. yes

20. Have you ever had severe pain in the middle of your chest lasting a half hour or more?

1. no

2. yes -> has a doctor ever told you that you have myocardial infarct?

    1. no

    2. yes →when and in which hospital has it been treated? ___________

21. Have you ever been told by the doctor that you have had? (circle also if you answer no)

	no	yes
chronic bronchitis………………………………………	1	2
emphysema………………………………………………	1	2
asthma……………………………………………………	1	2
allergic cold e.g. hay fever………………………	1	2
allergic excema…………………………………………	1	2
high blood pressure…………………………………	1	2
angina pectoris, in other words chest pain due to coronary disease…………………………	1	2
myocardial infarct “a coronary necrosis” in the breast muscle”…………………………………	1	2
diabetes……………………………………………………	1	2
peptic ulcers (stomach or duodenal ulcer)	1	2
rheumatoid arthritis………………………………	1	2
joint degeneration or arthrosis……………….	1	2
ischias…………………………………………………….	1	2
cataract……………………………………………………	1	2
glaucoma…………………………………………………	1	2
macular degeneration…………………………….	1	2
migraine………………………………………………….	1	2
epilepsy……………………………………………………	1	2
brain injury………………………………………………	1	2
meningitis…………………………………………………	1	2
other neurological disease………………………	1	2
long term liver disease……………………………	1	2
long term kidney disease…………………………	1	2
malignant tumor/cancer…………………………	1	2
any other long term or serious illness, which?	1	2

………

22. During the last year on how many days together have you used the following types of medicines (circle also, even if you have not used any)?

Alternatives 1 – 5 (not used, less than 10 days, 10-59 days, 60-180 days, more than 180 days)

	not used	less than 10 days	10-59 days	60-180 days	more than 180 days
pain relievers	1	2	3	4	5
antihypertensive drugs	1	2	3	4	5
heart drugs	1	2	3	4	5
antacids	1	2	3	4	5

drugs for skin disorders	1	2	3	4	5
eye drops	1	2	3	4	5
allergy drugs	1	2	3	4	5
sleeping pills	1	2	3	4	5
Tranquilizers	1	2	3	4	5
vitamin preparations	1	2	3	4	5
minerals	1	2	3	4	5
natural medicines	1	2	3	4	5

23. Have you ever had an accident due to falling asleep?

1. no
2. once
3. 2-5 times
4. more than 5 times

24. During the last 5 years have you had to visit a doctor or been hospitalized because of accident?

1. no
2. once
3. 2-5 times
4. 5 times or more

25. During the last year how many times you have had the following symptoms? (circle also, even if you did not have any)

	not at all	daily or almost daily	3-5 times a week	1-2 times a week	once a month	less often
stiffness in the limbs and body in the morning	1	2	3	4	5	6
stiffness in the limbs and body in the evening	1	2	3	4	5	6
pain and stiffness in the neck	1	2	3	4	5	6
white finger in the cold	1	2	3	4	5	6

sensitive areas on the neck, back, body or limbs	1	2	3	4	5	6
feeling of numbness in the limbs	1	2	3	4	5	6
burning eyes	1	2	3	4	5	6
dry mouth	1	2	3	4	5	6
headaches	1	2	3	4	5	6
stomach pains	1	2	3	4	5	6
flatulence	1	2	3	4	5	6
diarrhea	1	2	3	4	5	6
fatigue during the day	1	2	3	4	5	6

26. Do you have stiffness of limbs and body in the mornings?

1. no
2. yes, less than 15 min
3. yes, less than 30 min
4. yes, 1-2 hours
5. yes, more than 2 hours

27. Does the depression (rain, snowstorm, storm) affect your limb or body ache?

1. I do not have any
2. the change in weather does not affect the ache
3. the change in weather clearly increases the ache

28. During the last month have you had any of the following symptoms?

	daily or almost daily	about once a week	less often	not at all
heart palpitations without any physical efforts	1	2	3	4
irregular heart beats	1	2	3	4
chest pain while angry or emotionally upset	1	2	3	4

perspiration without any physical effort	1	2	3	4
facial blushing	1	2	3	4
shaking hands	1	2	3	4
voice trembling	1	2	3	4
muscle twitching	1	2	3	4

29. Have you ever experienced the following sudden symptoms when laughing, cheering, being excited or angry?

	never	1-5 times in a life	monthly	weekly	daily or almost daily
polvien pettämistä??	1	2	3	4	5
suun auki loksahtamista??	1	2	3	4	5
head nodding	1	2	3	4	5
maahan lyhistymistä??	1	2	3	4	5
feeling of powerlessness in the hands and feet	1	2	3	4	5

30. How tall are you?

_________________to the nearest centimeter

31. How much do you weight? (without any clothes)

_________________to the nearest kilo

32. What has been your highest weight (in females pregnancy not included)? _______________________kg

33. How much did you weight 12 months ago? _________________________________kg

34. How much did you weight 5 years ago? ____________________________________kg

35. How much did you weight when you were 30 years old? ___________________________kg

36. How much did you weight when you were 20 years old? ___________________________kg

37. How much your weight has varied by season (eg summer to winter) over the last five years?

1. less than 2kg
2. 2-5kg
3. 5-9kg
4. 10kg or more

38. Have you ever lost weight or tried to lose weight?

1. no
2. 1-2 times
3. 3-5 times
4. 6-10 times
5. more than 10 times

39. If you are married or cohabiting, what is the height and weight of your spouse?

_________________________cm_________________________________kg

40. How has your weight been compared to your co-twin at different ages?

a) at the age of 10 I was

1. thinner
2. about the same weight
3. fatter
4. I can't say

b) at the age of 20 I was

1. thinner
2. about the same weight
3. fatter
4. I can't say

c) at the age of 30 I was

1. thinner
2. about the same weight
3. fatter
4. I can't say

d) today I am

1. thinner
2. about the same weight
3. fatter
4. I can't say

41. What kind of physique you have compared to your co-twin?

1. I'm more muscular than my co-twin
2. we are similar in physique
3. my co-twin is more muscular than me
4. I can’t say

42. What kind of physique you have compared to other people?

1. I’m more muscular
2. I’m definitely more muscular
3. my muscles are normal in size
4. my muscles are somewhat thinner
5. my muscles are clearly thinner

43. Did you and your co-twin have the same size feet at the age of 20?

1. the same shoes fit quite well
2. his shoes were too small for me
3. his shoes were too big for me
4. I can’t say

44. What is your collar number (= neck circumference in cents) today?

1. 35 tai alle
2. 36-37
3. 38-39
4. 40-41
5. 42-43
6. 44-45
7. 46-47
8. 48 tai yli
9. I don’t know

45. What type of pants do you wear today?

1. slim fit
2. normal
3. stocky
4. very stocky

46. Have you in your entire life smoked more than 5-10 packs of cigarettes?

1. no-> go to Q50
2. yes

47. Do you smoke or have you at some time smoked regularly, in the other words daily or almost daily?

1. no-> go to Q50
2. yes

48. How old were you when you began to smoke regularly?

…………………………….years

49. Do you still smoke regularly?

1. No-> How old were you when you stopped smoking

……………………………..years

How many cigarettes did you smoke on average per day before you stopped?

1. none
2. less than 5
3. 5-9
4. 10-14
5. 15-19
6. 20-24
7. 25-39
8. more than 40

2. Yes-> How many cigarettes do you smoke daily on average?

1. none
2. less than 5
3. 5-9
4. 10-14
5. 15-19
6. 20-24
7. 25-39
8. more than 40

50. Have you in your entire life smoked more than 50-75 cigars/cigarillos, or more than 3-5 packs of Pipe tobacco?

1. no-> go to Q52
2. yes

51. Do you smoke or have you at some time smoked cigars, cigarillos or pipe regularly i.e. daily or almost daily?

1. no
2. I have quitted smoking daily
3. yes

52. In total, how long time have you been not smoking (smoking cessation) since you started smoking regularly? Count together all the periods you have not been smoking.

1. I have never quitted smoking
2. less than 6 months
3. 2-4 years
4. 5-9 years
5. 10 years or more
6. I can’t say
7. I’m not a smoker

53. Are you primarily

1. working outside home
2. working at home
3. at disability pension or sickness pension
4. at old age pension
5. student
6. unemployed, looking for a job
7. other, what ____________

54. At the present moment are you

1. working for somebody else on a monthly or hourly salary basis
2. working for somebody on a contractual basis
3. self-employed (non-farm)
4. farmer
5. I am not working at the present moment
6. I have never worked

55. Is your present work, or the work which you last did, in your opinion

1. very monotonous
2. quite monotonous
3. quite varying
4. very varying
5. I can't say
6. I have never worked

56. How much can you determine the pace of work in your present work?

1. I can determine the pace quite freely
2. I can influence it quite freely
3. I am doing work at a forced or nearly forced pace
4. I'm not working

57. How much can you determine your way of working in your present work?

1. I am quite free to choose my own way of working
2. I can determine my working methods to some extent
3. I can't determine my way of working
4. I'm not working

58. Is your present work or the work which you last did (mainly)

1. regular day work
2. regular night work
3. two-shift work without a night shift
4. two-shift work with a night shift
5. three-shift work
6. I have never worked

59. What kind of work do/did you do? The present work or the work which you last did?

1. mainly sedentary work, which requires very little physical activity
2. work which involves standing and walking, but no other physical activity
3. work which in addition to standing and walking requires lifting and carrying
4. heavy physical work

60. How many hours on average do you work per week?

1. I’m not working
2. less than 20 hours
3. 20-29 hours
4. 30-39 hours
5. 40-44 hours
6. 45-54 hours
7. 55 hours or more

61. How many hours on average do you do housework per week?

1. not any
2. less than 30 min per day
3. less than 1 hour per day
4. 1-3 hours per day
5. 4-5 hours per day
6. 6 hours or more

62. How many hours do you usually sleep at night?

1. 6 hours or less
2. 6,5 hours
3. 7 hours
4. 7,5 hours
5. 8 hours
6. 8,5 hours
7. 9 hours
8. 9,5 hours
9. 10 hours or more

63. How many hours of sleep do you usually need in order to be alert and active the next day?

1. 6 hours or less
2. 6,5 hours
3. 7 hours
4. 7,5 hours
5. 8 hours
6. 8,5 hours
7. 9 hours
8. 9,5 hours
9. 10 hours or more

64. How often do you suffer from insomnia?

1. every night or nearly every night
2. 3 – 5 nights in a week
3. 1 – 2 nights in a week
4. less than one night in a week
5. never or less than once in a month

65. Are you having trouble falling asleep in the evenings?

1. never or less than once in a month
2. less than one night in a week
3. 1 – 2 nights in a week
4. 3 – 5 nights in a week
5. every evening or nearly every evening

66. How fast do you usually fall asleep in the evenings?

1. in less than 10 min
2. in 10-20 min
3. in 21-30 min
4. in 31-40 min
5. in over 40 min

67. Have you had any unusual experiences with falling asleep or waking up e.g you have seen, heard or felt something that did not really exist?

1. never
2. less than once a month
3. less than once a week
4. 1-2 times a week
5. 3-5 times a week
6. daily or even many times a day

68. On average, how many times do you wake up at night?

1. I don’t usually wake up at night
2. a couple nights a week
3. once a night
4. twice a night
5. 3-4 times a night
6. 5 or more

69. Can you sleep in peace?

1. usually
2. sometimes I can’t
3. repeatedly no

70. The following is a set of statements that describe your feelings right now. Circle the option that best describes your feelings right now**. *(Beck's Depression Inventory)***

1. I do not feel sad.
2. I feel sad
3. I am sad all the time and I can't snap out of it
4. I am so sad and unhappy that I can't stand it.

1. I am not particularly discouraged about the future.
2. I feel discouraged about the future.
3. I feel I have nothing to look forward to.
4. I feel the future is hopeless and that things cannot improve.

1. I do not feel like a failure.
2. I feel I have failed more than the average person.
3. As I look back on my life, all I can see is a lot of failures
4. I feel I am a complete failure as a person.

1. I get as much satisfaction out of things as I used to.
2. I don't enjoy things the way I used to.
3. I don't get real satisfaction out of anything anymore.
4. I am dissatisfied or bored with everything.

1. I don't feel particularly guilty
2. I feel guilty a good part of the time.
3. I feel quite guilty most of the time.
4. I feel guilty all of the time.

1. I don't feel I am being punished.
2. I feel I may be punished.
3. I expect to be punished.
4. I feel I am being punished.

1. I don't feel disappointed in myself.
2. I am disappointed in myself
3. I am disgusted with myself.
4. I hate myself.

1. I don't feel I am any worse than anybody else.
2. I am critical of myself for my weaknesses or mistakes.
3. I blame myself all the time for my faults.
4. I blame myself for everything bad that happens

1. I don't have any thoughts of killing myself.
2. I have thoughts of killing myself, but I would not carry them out.
3. I would like to kill myself.
4. I would kill myself if I had the chance.

1. I don't cry any more than usual.
2. I cry more now than I used to.
3. I cry all the time now.
4. I used to be able to cry, but now I can't cry even though I want to.

1. I am no more irritated by things than I ever was.
2. I am slightly more irritated now than usual.
3. I am quite annoyed or irritated a good deal of the time.
4. I feel irritated all the time.

1. I have not lost interest in other people.
2. I am less interested in other people than I used to be.
3. I have lost most of my interest in other people.
4. I have lost all of my interest in other people.

1. I make decisions about as well as I ever could.
2. I put off making decisions more than I used to.
3. I have greater difficulty in making decisions more than I used to.
4. I can't make decisions at all anymore.

1. I don't feel that I look any worse than I used to.
2. I am worried that I am looking old or unattractive.
3. I feel there are permanent changes in my appearance that make me look unattractive
4. I believe that I look ugly.

1. I can work about as well as before.
2. It takes an extra effort to get started at doing something.
3. I have to push myself very hard to do anything.
4. I can't do any work at all.

1. I can sleep as well as usual.
2. I don't sleep as well as I used to.
3. I wake up 1-2 hours earlier than usual and find it hard to get back to sleep.
4. I wake up several hours earlier than I used to and cannot get back to sleep.

1. I don't get more tired than usual.
2. I get tired more easily than I used to.
3. I get tired from doing almost anything.
4. I am too tired to do anything.

1. My appetite is no worse than usual.
2. My appetite is not as good as it used to be.
3. My appetite is much worse now.
4. I have no appetite at all anymore.

1. I haven't lost much weight, if any, lately.
2. I have lost more than 5 kilos.
3. I have lost more than 4 kilos.

4. I have lost more than 6 kilos

1. I am no more worried about my health than usual.
2. I am worried about physical problems like aches, pains, upset stomach, or constipation
3. I am very worried about physical problems and it's hard to think of much else.
4. I am so worried about my physical problems that I cannot think of anything else.

1. I have not noticed any recent change in my interest in sex.
2. I am less interested in sex than I used to be.
3. I have almost no interest in sex.
4. I have lost interest in sex completely.

71. How much of the following alcoholic beverages do you drink on average?

Beer

1. never
2. less than a bottle a week
3. 1-4 bottles a week
4. 5-12 bottles a week
5. 13-24 bottles a week
6. 25-47 bottles a week
7. more than 48 bottles a week

Wine or other mild alcohol beverages

1. never
2. less than a glass a week
3. a glass to 4 glasses a week
4. 1-2,5 bottles a week
5. 3-4,5 bottles a week
6. 5-9 bottles a week
7. more than 10 bottles a week

Hard liquor

1. never
2. less than a half bottle a month
3. a half bottle to a bottle and a half a month
4. 2- 3,5 bottles a month
5. 4-9 bottles a month
6. 10-19 bottles a month
7. more than 20 bottles a month

72. How often do you use alcohol? Which of the following alternatives best describes your use of beer, wine and hard liquor?

	never	on less than two days a month	on 3-8 days a month	on 9-16 days a month	over 16 days a month
Beer	1	2	3	4	5
Wine	1	2	3	4	5
Liquor	1	2	3	4	5

73. Does it happen that at least once a month and on the same occasion you drink more than five bottles of beer or more than bottle of wine or more than half a bottle of hard liquor?

1. no
2. yes

74. Have you ever passed out while using the alcohol during the last year?

1. not once
2. once
3. two-three times
4. four-six times
5. seven times or more

75. In following we ask about physical activity during leisure time or on the way to work over the past 12 months. First, evaluate what kind of strain you are exercising. Then estimate the average number of hours per week that you exercise for each level.

	not at all	less than 30 minutes per week	30 minutes to 1 hour per week	2-3 hours per week	4 hours or more per week
walking	1	2	3	4	5
alternatively jogging and walking	1	2	3	4	5
jogging	1	2	3	4	5
running	1	2	3	4	5

76. How well do the following statements describe you? Choose the best alternative. The statements describes you very well, well, not very well, not at all

The statement describes me

	very well	well	not very well	not at all
In general I am unusually tense or nervous	1	2	3	4
There is a great deal of strain connected with my daily activities	1	2	3	4
At the end of the day I am completely exhausted mentally and physically	1	2	3	4
My daily activities are extremely trying and stressful	1	2	3	4

77. In the following a number of events that can happen in life are listed. Have any of these happened to you?

the event happened to me

	Not at all	During the last six months	During the last five years	Earlier
death of spouse	1	2	3	4
death of close relative or good friend	1	2	3	4
marked change in the health of family member	1	2	3	4
difficulties of the a sexual nature	1	2	3	4
marked difficulties with superiors, colleagues or subordinates at work	1	2	3	4
significant difficulties in the economic situation	1	2	3	4
divorce or separation	1	2	3	4
brake in long term human relationship (no divorce)	1	2	3	4

loss of job	1	2	3	4
marked increase in difficulties with spouse (not divorce)	1	2	3	4
disease or injury causing over three weeks work disability	1	2	3	4
another hard-hit event	1	2	3	4
remarkably positive change in working life	1	2	3	4
remarkably positive change in relationship	1	2	3	4
some other remarkable change in general in life	1	2	3	4

78. How many people you meet or speak with during the ordinary week?

1. none
2. 1-2
3. 3-5
4. 6-10
5. 11-15
6. > 15

79. How many friends you have that can come to your home at any time and feel at home. They would not be disturbed even if your home was not cleaned or you were just eating.

1. none
2. 1-2
3. 3-5
4. 6-10
5. 11-15
6. > 15

80. How many friends or family members you have, whom you can talk directly and openly?

1. none
2. 1-2
3. 3-5
4. 6-10
5. 11-15
6. > 15

81. Do you know any person, from whom you can get a friend’s support?

1. no
2. yes

82. Do you have anyone with whom you can share your inmost feelings and to whom you can confide in?

1. no
2. yes

83. Every human being has a certain idea about himself. We ask you to describe with the words presented what kind of person you think you are. The words are opposites and present traits and characteristics as far away from each other than possible. In between the words you can find five lines. Circle one of the lines between the words, nearer by the word you think that better describes you

never late	casual about appointments
not competitive	very competitive
open	reserved
rushed	not rushed
takes thing one at time	tries to do many thing at time
rarely quarrelsome	quarrelsome
fast	slow
“sits” on feelings	expresses feelings
many interests	few interests
does not get angry easily	gets angry easily
determined	uncertain
communicative	uncommunicative
submissive	dominating
self-confident	not sure of oneself
always honest	sometimes dishonest in little things
quiet	talkative
diligent	lazy
gets easily irritated	does not get irritated
does not always tell the whole truth	tells always the whole truth

84. Do you feel that your life at the present moment is very interesting, fairly interesting, fairly boring or very boring?

1. very interesting
2. fairly interesting
3. fairly boring
4. very boring
5. don’t know

85. Do you feel that at the present moment your life is very happy, fairly happy, fairly sad or very sad very happy?

1. very happy
2. fairly happy
3. fairly sad
4. very sad
5. don’t know

86. Do you feel that at the present moment your life is very easy, fairly easy, fairly hard or very hard?

1. very easy
2. fairly easy
3. fairly hard
4. very hard
5. don't know

87. Do you feel that at the present moment you are very lonely, fairly lonely or not at all lonely?

1. very lonely
2. fairy lonely
3. not at all lonely
4. don't know

88. Do you snore in your sleep (if necessary, ask another person)?

1. every night or almost every night
2. 3-5 nights a week
3. 1-2 nights a week
4. less than 1 night a week
5. less than once a month
6. I don't know

89. How many years have you snored?

1. I don't snore
2. I don't know
3. 1-4 years
4. 5-8 years
5. 9-12 years
6. 13-19 years
7. 20 years or more

90. How does your snoring sound (described by another person)?

1. I don't snore
2. I snore quietly
3. I snore loudly and steadily
4. I snore loudly and unevenly

91. Whether your alcohol consumption affects your snoring?

1. I snore only after drinking alcohol
2. I also snore without alcohol
3. I don't snore even though I have used alcohol
4. I don't use alcohol
5. I don't know

92. Has anyone noticed that you have more than 10 seconds of apnea in your sleep (interruption of breath)?

1. every night or nearly every night
2. 3 – 5 nights in a week
3. 1 – 2 nights in a week
4. less than once a week
5. less than once a month or never
6. I don’t know

93. Do you sweat so much when sleeping that your sleepwear, pillowcase or sheets get wet?

1. every night or nearly every night
2. 3 – 5 nights in a week
3. 1 – 2 nights in a week
4. less than one night in a week
5. less than once a month or never

94. How often did you have the following symptoms in childhood or school age?

	weekly	about once a month	less often	never	I don’t know
sleepwalking	1	2	3	4	5
sleep talking	1	2	3	4	5
teeth grinding	1	2	3	4	5
bedwetting over 4 years old	1	2	3	4	5
nightmares	1	2	3	4	5
night terrors	1	2	3	4	5

95. As an adult have you had the following symptoms?

	weekly	about once a month	less often	never	I don’t know
sleepwalking	1	2	3	4	5
sleep talking	1	2	3	4	5
teeth grinding	1	2	3	4	5
bedwetting	1	2	3	4	5
nightmares	1	2	3	4	5
night terrors	1	2	3	4	5

96. Do you feel yourself tired in the morning when you wake up?

1. every morning or nearly every morning
2. 3 - 5 mornings in a week
3. 1 - 2 mornings in a week
4. less than once a week
5. less than once a month or never

97. Do you feel tired during the daytime?

1. every day or nearly every day
2. 3 - 5 days per week
3. 1 - 2 days per week
4. less than once a week
5. less than once a month or never

98. Do you sleep during the day time (do you take a nap)

1. don’t need to
2. I would like to but I can’t
3. twice a week or less
4. 3-5 times a week
5. daily or almost daily

99. Do you have unintentional falling asleep during the day time?

	never	monthly or less often	weekly	daily	many times a day
while reading	1	2	3	4	5
while travelling by train, bus etc.	1	2	3	4	5
while standing in line	1	2	3	4	5
while eating	1	2	3	4	5
In another situation where people don't usually fall asleep	1	2	3	4	5

100. Has your doctor prescribe you medicine for narcolepsy (e.g. Bonumin, Mirapront, Catovit, Reactivan, Dexedrin, Teronac, Ritalin..)

1. no
2. yes-> which_____________________________________

101-103 The Anger Expression Scale (AX) is a 24-item self report scale which yields four scores (Spielberger ***et*** al., 1985 1) express anger (Anger-Out); 2) experience but hold in angry feelings (Anger-In); and 3) control the experience and expression of anger (Anger-Control).

Millainen minä yleensä olen?

	ei sovi juuri koskaan	sopii joskus	sopii melko usein	sopii aina
1. Suutun helposti	.. l	2	3	4
2. Kiivastun herkästi	.. l	2	3	4
3. Minua suututtaa jos en saa tunnustusta hyvin tekemästäni työstä	1 ..	2	3	4
4. Kun suutun, niin minun tekee mieli sanoa toisille ilkeästi	.. 1	2	3	4
5. Jos minulle on aiheutettu oikein paljon harmia, niin minun tekisi mieli läimäyttää jotakuta	.. 1	2	3	4
6. Minua raivostuttaa, kun hyvää aikaansaannostani haukutaan huonoksi	.. l	2	3	4
