# Supplementary material for: Changes in self-reported sleep duration with age - a 36-year longitudinal study of Finnish adults
Source: BMC Public Health. 2020 Sep 9;20:1373. doi: 10.1186/s12889-020-09376-z (PMC7487757; doi:10.1186/s12889-020-09376-z)
Supplement: Supplementary file 4 — Additional file 4:. FTC old cohort 2011 wave 4 questionnaire English translation. Unofficial translation of mailed questionnaire used in wave 4 of the older Finnish Twin Cohort in 2011, originals in Finnish and Swedish. [file 12889_2020_9376_MOESM4_ESM.pdf]

TWIN RESEARCH, OLDER TWINS, 4. QUESTIONNAIRE Q2011

1. How long did you live with your twin partner/co-twin? (-81-versiossa käytetty twin partner)
    1. I am still living with him/her
    2. We have lived together until the age of ___ years.

2. How often do you meet or communicate to your twin partner nowadays?
    1. daily
    2. about once a week
    3. about once a month
    4. about once every six month
    5. less often/less frequently
    6. never

3. Are you
    1. single
    2. married
    3. re-married
    4. living with somebody, but unmarried
    5. divorced or separated
    6. widow/widower

NEXT QUESTIONS ARE ABOUT YOUR HEALTH

4. What do you think about your health status? Is it at the moment:
    1. very good
    2. fairly good
    3. average
    4. fairly poor
    5. poor

5. When was the last time your blood pressure has been measured?

    6. less than half year ago
    7. half year – less than 1 year ago
    8. 1 year – less than 5 years ago
    9. 5 years or more than 5 years ago
    10. never

6. Has a doctor (or other health professional) ever told you that you have arterial hypertension or elevated blood pressure?
    a. no

   b. yes

7. When was the last time that the fat content of your blood (cholesterol) has been determined?
   a. less than half a year ago
   b. half year – less than 1 year ago
   c. 1 year – less than 5 years ago
   d. 5 years or more than 5 years ago
   e. never
   f. I don't know

8. Do you use any prescription medicine nowadays to lower your cholesterol? or: Are you currently using any prescription medicine to lower your blood cholesterol?
   a. no
   b. yes

9. Has the fat content (overall cholesterol) of your blood been determined/measured during the past five years?
   a. no
   b. I don't know
   c. yes and the value was:
      1. below 4.0
      2. 4.0 - 4.9
      3. 5.0 – 5.9
      4. 6.0 – 6.9
      5. 7.0 – 7.9
      6. 8.0 or more

10. When was the last time your blood sugar has been determined/measured?
   a. less than half a year ago
   b. half year – less than 1 year ago
   c. 1 year – less than 5 years ago
   d. 5 years or more than 5 years ago
   e. never
   f. I don't know

11. Have you ever been told by a doctor that you have diabetes?
(You can circle more alternatives if necessary)
   a. no
   b. no, but elevated blood sugar concentration or latent diabetes
   c. yes, diabetes during pregnancy
   d. yes, type 1 or juvenile diabetes
   e. yes, type 2 or adult diabetes
   f. yes, but I don't know the type

12. Do you regularly or for extended periods of time have a cough?
    a. no.......go to question 15
    b. yes
13. How many months in a row do you cough per year?
    a. less than three months in a row
    b. more than three months in a row
14. For how many months in a row do you bring up phlegm from your chest per year?
    a. less than three months in a row
    b. more than three months in a row
15. Do you usually get short of breath when you walk uphill, climb stairs or hurry on level grounds?
    a. no
    b. yes
16. Do you usually get short of breath when walking on level ground at an ordinary pace with people of your own age?
    a. no
    b. yes
17. Do you have to stop to breathe because of shortness of breath when you walk at your own pace on level ground 150 meters?
    a. no
    b. yes
18. Do you usually get short of breath when standing still, for example when dressing or washing?
    a. no
    b. yes
19. Have you ever had severe pain in the middle of your chest lasting a half hour or more?
    a. no
    b. yes -> has a doctor ever told you that you have myocardial infarct?
        i. no
        ii. yes →when has it been treated? ___________(year)

20. During the last years have you had pains in the back, shoulders or neck that make it difficult for you to work (circle also if not)?
    a. pain in the back no 1 yes 2
    b. pain in the shoulders no 1 yes 2
    c. pain in the neck no 1 yes 2

21. Have you noticed or have you been told (by a person who sleeps with you) that you grind or grit your teeth together while sleeping?
    a. every night or nearly every night
    b. 3 – 5 nights per week
    c. 1 – 2 nights per week

d. less than one night per week
e. never or less than once a month

22. Do you grit your teeth together when you are awake?
    a. every day or nearly every day
    b. 3 – 5 days per week
    c. 1 – 2 days per week
    d. less than one day per week
    e. never or less than once a month
23. Have you ever been told by a doctor that you have or have had (circle also if you answer no) No----1 Yes----2
    A. bronchial asthma
    B. emphysema
    C. chronic bronchitis
    D. chronic obstructive pulmonary disease (COPD)
    E. allergic fever e.g. hay fever
    F. coronary disease
    G. cerebral palsy/stroke (cerebral hemorrhage or cerebral infarction)
    H. dementia, including Alzheimer's disease
    I. depression
    J. migraine
    K. rheumatoid arthritis
    L. joint degeneration or arthrosis
    M. fibromyalgia
    N. Parkinson's disease
    O. any other serious or long-term illness, which? ___________________

24. During the last year on how many days together have you used the following types of medicines (circle also, even if you have not used any)?

Alternatives 1 – 5 (not used, less than 10 days, 10-59 days, 60-180 days, more than 180 days)

A. fortifying medicines (like iron or vitamin preparations)
B. pain relievers
C. antihypertensive drugs
D. heart drugs
E. allergy drugs
F. asthma drugs
G. antacids
H. sleeping pills
I. sedatives

J. antidepressants

NEXT TWO QUESTIONS ARE ABOUT HORMONE REPLACEMENT THERAPY

Questions 25 and 26 are to be answered by women only. Men can go to question 27.

25. During the last month, have you used hormone replacement therapy as pills, gel or plasters?
    a. no
    b. yes
26. How long have you been using hormone replacement therapy together?
    a. I have never used
    b. less than a year
    c. 1 – 4 years
    d. 5 years or more

NEXT QUESTIONS ARE ABOUT SLEEP

27. How many hours do you usually sleep at night? (alternatives)
28. Do you generally sleep well?(alternatives)
29. Do you snore in your sleep?(alternatives)
30. Has anyone noticed that you have more than 10 seconds of apnea in your sleep (interruption of breath)?(alternatives)
31. How often do you suffer from insomnia?(alternatives)
32. Try to estimate to what extent you are a “morning person” or an “evening person”
    a. I am clearly a morning person (bright in the morning and sleepy in the evening.
    b. I am somewhat a morning person.
    c. I am somewhat an evening person (sleepy in the morning and bright in the evening).
    d. I am clearly an evening person.

NEXT QUESTIONS ARE ABOUT WORK

33. Are you primarily
    a. working outside home
    b. working at home
    c. at old age pension
    d. at disability pension or sickness pension?
    e. unemployed, looking for a job

f. other, what _____________

34. At the present moment are you
    a. working for somebody else on a monthly or hourly salary basis
    b. working for somebody on a contractual basis
    c. self-employed (non-farm)
    d. farmer
    e. I am not working at the present moment
    f. I have never worked
35. Is your present work, or the work which you last did, in your opinion
    a. very monotonous
    b. quite monotonous
    c. quite varying
    d. very varying
    e. I can't say
    f. I have never worked
36. Is your present work or the work which you last did (mainly)
    a. regular daywork
    b. regular nightwork
    c. two-shift work without a night shift
    d. two-shift work with a night shift
    e. three-shift work
    f. I have never worked
37. What kind of work did/do you do? The present work or the work which you last did?
    a. mainly sedentary work, which requires very little physical activity
    b. work which involves standing and walking, but no other physical activity
    c. work which in addition to standing and walking requires lifting and carrying
    d. heavy physical work
38. How much can you determine the pace of work in you present work?
    a. I can determine the pace quite freely
    b. I can influence it somewhat
    c. I am doing work at a forced or nearly forced pace
    d. I am not working

NEXT QUESTIONS ARE ABOUT PENSION INTENTIONS (PLANS)

39. If you had the possibility to choose between continuing your work and going over to pension, what would you do?
    a. I would continue working
    b. I would go over to pension
    c. I can't say
    d. I have retired

40. Have you considered to apply for disability– pension, individual early retirement, employment pension or some other pension?
    a. I have not considered to apply for a pension
    b. applying for a pension has crossed my mind
    c. I have considered seriously to apply for a pension
    d. I have applied for a pension
    e. I have retired already

QUESTIONS ABOUT SMOKING

41. Have you in your entire life smoked more than 100 cigarettes (5 packs)?(-81-kysely)/ Over your lifetime, have you smoked more than.... (nico)
    a. no I haven't
    b. yes

42. When is the last time you have been smoking?
    a. yesterday or today
    b. 2 days – less than 1 month ago
    c. 1 month – less than half a year ago
    d. half a year – less than a year ago
    e. a year – less than 5 years ago
    f. 5 years – less than 10 years ago
    g. 10 years – less than 20 years ago
    h. 20 years ago or earlier
    i. I have never smoked
43. Do you smoke or have you at some time smoked regularly, in other words daily or almost daily?
    a. no---→ go to Q48 page 14
    b. yes
44. Do you still smoke cigarettes regularly?
    a. no---→How old were you when you stopped smoking?
    ______________ years
    How many cigarettes did you smoke on average daily before you stopped?

1	none
2	less than 5 cigarettes
3	5 – 9 cigarettes
4	10 – 14 cigarettes
5	15 – 19 cigarettes
6	20 – 24 cigarettes
7	25 – 39 cigarettes
8	more than 40 cigarettes

    b. yes-→ How many cigarettes do you smoke daily on average?

1. none
2. less than 5 cigarettes
3. 5 – 9 cigarettes
4. 10 – 14 cigarettes
5. 15 – 19 cigarettes
6. 20 – 24 cigarettes
7. 25 – 39 cigarettes
8. more than 40 cigarettes

45. What is the largest amount of cigarettes that you have ever smoked in any 24 hours time?

    ____________ cigarettes

46. Think about the time when you were smoking the most. Please answer even if you have quit smoking.
    a. How soon after you wake up do you smoke your first cigarette?
        i. within 5 minutes
        ii. 6-30 minutes
        iii. 31-60 minutes
        iv. after 60 minutes
    b. Do/did you find it difficult to refrain from smoking in places where it is forbidden?
        i. no
        ii. yes
    c. Which cigarette would you hate/have hated most to give up?
        i. first cigarette in the morning
        ii. some other cigarette
    d. How many cigarettes per day do/did you smoke?
        i. 1-10 cigarettes
        ii. 11-20 cigarettes
        iii. 21-30 cigarettes
        iv. 31 cigarettes or more
    e. Do/did you smoke more frequently during the first hours after waking up than during the rest of the day?
        i. no
        ii. yes
    f. Do/did you smoke when you are/were so ill that you are/were in bed most of the day?
        i. no
        ii. yes

47. Here you have some statements concerning smoking. Under every statement, please circle the most suitable alternative for you within scale 1-7. When you answer the statements, think about the time when you have been smoking the most. Please answer even if you have quitted smoking.

1= not at all true in my case 7=quite true in my case

A. I often smoke without thinking about it.
B. Cigarettes control me.
C. I usually want to smoke right after I wake up.
D. It’s hard to ignore an urge to smoke.
E. The flavor of a cigarette is pleasing.
F. Smoking helps me feel better in seconds.
G. I smoke without deciding to.
H. I frequently light cigarettes without thinking about it.
I. Most of my daily cigarettes taste good.
J. Sometimes I feel like cigarettes rule my life.
K. I frequently crave cigarettes.
L. Some of the cigarettes I smoke taste great.
M. I’m really hooked on cigarettes.
N. My urges to smoke keep getting stronger if I don’t smoke.
O. I find myself reaching for cigarettes without thinking about it.
P. Other smokers would consider me a heavy smoker.
Q. When I haven’t been able to smoke for a few hours, the craving gets intolerable.
R. I smoke within the first 30 minutes of awakening in the morning.
S. Smoking really helps me feel better if I’ve been feeling down.
T. My smoking is out of control.
U. I consider myself a heavy smoker.
V. Even when I feel good, smoking helps me feel better.

48. Do you smoke or have you at some time smoked cigars, cigarillos or pipe regularly, i.e. daily or almost daily?
    a. I have never smoked daily
    b. I have stopped smoking regularly
    c. I smoke daily
49. Have you during the past year (12 months) used nicotine replacement therapy (gum, patch, lozenge etc.)?

    1 No, I have not used
    2 Yes, I have used as a smoking cessation aid
    3 Yes, I have used for another reason

50. At your home, how many years together has someone been smoking indoors daily or almost daily?
    a. none
    b. 1 – 10 years
    c. 11 – 20 years
    d. 21 – 30 years

e. over 30 years

51. At your work premises, how many years together has someone been smoking indoors daily or almost daily?
    a. none
    b. 1 – 10 years
    c. 11 – 20 years
    d. 21 – 30 years
    e. over 30 years
52. On an average how many hours per day are/were you at your work in premises where there is/was cigarette smoke?
    a. none
    b. less than 1 hour
    c. 1 – 4 hours
    d. more than 4 hours

NEXT QUESTIONS ARE ABOUT USE OF ALCOHOL

53. At what age did you drink a whole glass of alcohol for the first time?
(E.g. a little bottle of beer, a glass of wine or a “shot”)

_________ years

0 I have never drank alcohol --→ go to question 60

54. How often do you drink following types of alcohol on average?
    a. Beer:
        i. never
        ii. less than a bottle per week
        iii. 1 – 4 bottles per week
        iv. 5 – 8 bottles per week
        v. 9 -12 bottles per week
        vi. 13 – 24 bottles per week
        vii. 25 – 47 bottles per week
        viii. more than 48 bottles per week

        GUIDELINE/note: one bottle means 0,33 litres of beer

    b. Wine or some other mild alcoholic beverages:
        i. never
        ii. less than a glass per week
        iii. 1 – 2 glasses per week
        iv. 3 –5 glasses per week
        v. 1 -2,5 bottles per week
        vi. 3 – 4,5 bottles per week
        vii. 5 – 9 bottles per week
        viii. more than 10 bottles per week

GUIDELINE/note: one bottle means 0,75 litres of wine or other mild alcoholic beverages

c. Liquor:
   i. never
   ii. less than half a bottle within a month
   iii. half – one and a half bottles within a month
   iv. 2 – 3,5 bottles within a month
   v. 4 – 9 bottles within a month
   vi. 10 – 19 bottles within a month
   vii. more than 20 bottles within a month
   GUIDELINE/note: one bottle liquor means 0,5 litres of hard liquor (e.g. Koskenkorva)

55. Does it happen that at least once in a month and on the same occasion you drink more than five bottles of beer or more than a bottle of wine or more than half a bottle of hard liquor?
   1. yes
   a. 2. no

56. Have you passed out while using alcohol during the last year?
   0 not once
   1 once
   2 two-three times
   4 four-six times
   7 seven times or more

57. Have you had hangover during the last year?
   0 not once
   1 once
   2 two-three times
   4 four-six times
   7 seven times or more

58. What is the largest number of drinks you have ever had in a 24-hour period? (even if this would have happened only once, e.g. at midsummer, 1st of May, on journeys, on cruises etc.) A best estimate is also accepted
   ____________ drinks (examples: 0.33 l mild beer = 1 drink
   0.5 l mild beer = 1,5 drinks, 0,5 l strong beer = 2 drinks, 12cl mild wine = 1 drink, a bottle of (0.75 l) mild wine =6 drinks, 4 cl strong liquor = 1 drink, a bottle (0.5 l) strong liquor = 13 drinks

59. Next, we would like to know more about habits and experiences connected with your current and previous use of alcohol

A. Have you ever had the habit of taking a drink before going to a party?
B. Have you ever had the habit of drinking a bottle of wine or corresponding amount of beer or other alcoholic beverages over the weekend?
C. Have you sometimes drunk a couple of drinks daily/a day to relax?
D. Have you ever had to drink more alcohol than before to get the same effect?
E. Have you ever had difficulties not drinking more than your friends?
F. Have you ever fallen asleep after moderate drinking without knowing how you got to bed?
G. Have you had a bad conscience after drinking sometimes?
H. Have you taken a drink (the day after a party) for your hang-over sometimes?
I. Have you sometimes tried to avoid alcoholic beverages for a determined period of time – e.g. a week?
J. Have you ever felt that it is difficult to stop after one drink after you have started drinking?
K. Have you ever felt that someone near to you or your workmate thinks that you should drink less alcohol?

60. How much do you usually drink a day of? (-81 kysely)/ How many cups of coffee or tea do you usually drink daily? If you do not drink daily, write 0.
   a. Coffee______ cups
   b. Tea________ cups

NEXT QUESTIONS ARE ABOUT PHYSICAL EXERCISE

61. How much of your daily journey to work is spent in walking, cycling, running and/or cross-country skiing in total?
   a. less than 15 minutes
   b. 15 minutes – less than half an hour
   c. half an hour – less than an hour
   d. an hour or more
   e. I am not presently at work
62. Physical activity during leisure time (activity which does not occur at work or on the way to work). Here are five alternatives to choose from when deciding on the exercise you get during your leisure time. Which one applies best to you when considering the exercise you get during the year as a whole?
   a. practically none
   b. a little
   c. a moderate amount
   d. quite a lot
   e. a great deal
63. How long does the physical activity during leisure time last at one session on average?
   a. less than 15 minutes

b. 15 minutes – less than half an hour
c. half an hour – less than an hour
d. an hour to less than two hours
e. over two hours

64. Presently how many times per month do you engage in physical activity during your leisure time?
   a. less than once a month
   b. 1-2 times a month
   c. 3-5 times a month
   d. 6-10 times a month
   e. 11-19 times a month
   f. more than 20 times a month

65. Is your physical activity during leisure time about as tiring on average as:
   a. walking
   b. alternatively walking and jogging
   c. jogging (light run)
   d. running

66. How many hours per day do you sit on average?
   a. in office or similar places (e.g. during a working day)
      i. less than an hour
      ii. an hour – less than two hours
      iii. two hours - less than four hours
      iv. four hours or more
   b. at home watching tv or videos
      1. less than an hour
      2. an hour – less than two hours
      3. two hours - less than four hours
      4. four hours or more
   c. at home at the computer
      1. less than an hour
      2. an hour – less than two hours
      3. two hours - less than four hours
      4. four hours or more
   d. in a vehicle
      1. less than an hour
      2. an hour – less than two hours
      3. two hours - less than four hours
      4. four hours or more
   e. elsewhere
      1. less than an hour
      2. an hour – less than two hours
      3. two hours – less than four hours
      4. four hours or more

67. In the following there is a list of events that can happen in life. Have any of these happened to you? (the event happened to me 1. not at all 2. during the last six months 3. during the last five years 4. earlier)
    a. Death of your spouse
    b. Death of your child
    c. Death of a close relative or a good friend
    d. Marked change in the health of a family member (not death)
    e. Difficulties of a sexual nature
    f. Marked difficulties with superiors, colleagues or subordinates at work
    g. Marked worsening in financial situation
    h. Divorce or separation
    i. Break in long-term human relationship (not divorce)
    j. Loss of job
    k. Marked increase in difficulties with spouse (not divorce)
    l. Disease or injury causing over three weeks work disability

   Has any of the following events happened to you? (same alternatives)

   13. Remarkable positive change at work
   14. Remarkable positive change in human relations
   15. Some other remarkable change in life in general

68. Has any of the following serious/exceptional events happened to you in your lifetime?
    A. A serious traffic accident
    B. Other serious accident
    C. Fire or catastrophe
    D. Hit or kicked hard enough to get injured
    E. Forced/tried to force to sexual contact
    F. A violence crime where a gun, a knife or some other weapon was used
    G. Parent's divorce or separation
    H. Some other very traumatic event, which_________________

In case you answered "yes" to any of the preceding questions, write the letter of the event that was the most shocking to you to the line above:_______

When this happened, did you feel very frightened, horrified or helpless
1 no 2 yes
When this happened, did you feel unreal, absent, disoriented or otherwise strange? 1 no 2 yes

Next questions are about human relations and mood

69. Do you know any person, from whom you can get a friend's support?
    a. no

    b. yes
70. Do you have anyone with whom you can share your inmost feelings and to whom you can confide in?
    a. no
    b. yes
71. When you think of your sexual life as a whole, would you say that it is
    a. very satisfactory
    b. fairly satisfactory
    c. no satisfactory nor unsatisfactory
    d. fairly unsatisfactory
    e. very unsatisfactory
72. Do you feel that your life at the present moment is very interesting, fairly interesting, fairly boring or very boring?
    a. very interesting
    b. fairly interesting
    c. fairly boring
    d. very boring
    e. don't know
73. Do you feel that at the present moment your life is very happy, fairly happy, fairly sad or very sad?
    a. very happy
    b. fairly happy
    c. fairly sad
    d. very sad
    e. don't know
74. Do you feel that at the present moment your life is very easy, fairly easy, fairly hard or very hard?
    a. very easy
    b. fairly easy
    c. fairly hard
    d. very hard
    e. don't know
75. Do you feel that at the present moment you are very lonely, fairly lonely or not at all lonely?
    a. very lonely
    b. fairly lonely
    c. not at all lonely
    d. don't know
76. Below is a list of the ways you might have felt or behaved. Please circle the alternative which best describes how often the statement corresponds your feelings and behavior during the past WEEK

(*Rarely or none of the time – 1, Sometimes or a little of the time – 2, Occasionally or a moderate amount of the time – 3, Most or all of the time – 4)*

    A. I was bothered by things that usually don't bother me

B. I did not feel like eating; my appetite was poor
C. I felt that I could not shake off the blues even with help from my family or friends
D. I felt that I was just as good as other people
E. I had trouble keeping my mind on what I was doing
F. I felt depressed
G. I felt that everything I did was an effort
H. I felt hopeful about the future
I. I thought my life had been a failure
J. I felt fearful
K. My sleep was restless
L. I was happy
M. I talked less than usual
N. I felt lonely
O. People were unfriendly
P. I enjoyed life
Q. I had crying spells
R. I felt sad
S. I felt that people disliked me
T. I could not get "going".

77. In the following we will present some questions that deal with the way people feel and act. For each question circle that alternative (no or yes) that best describes the way you generally feel and act. Circle the alternative which first comes into your mind.

A. Do you like to have lots of things going on around you?
B. Are you often uneasy, feeling that there is something you want without knowing what it is?
C. Do you almost always have an answer ready when spoken to?
D. Are you sometimes happy and sometimes sad without any special reason?
E. Do you prefer to keep to the background in the company of people?
F. Do you regard yourself as happy and carefree?
G. Do you often reach decisions too late?
H. Do you often feel tired and listless without any special reason?
I. Do you have a lively manner / Are you lively and talkative?
J. Can you describe your thoughts in words quickly?
K. Are you often lost in your thoughts (even if others expect you to participate in conversation)?
L. Do you have anything against selling lottery tickets or asking people for money for some purpose you value yourself?
M. Are you extremely sensitive in some respects?
N. Do you sometimes feel so restless that you cannot sit still?
O. Do you have difficulties in falling asleep in the evenings?
P. Do you usually keep your distance with others except your friends?

Q. Do you have any nervous problems?
R. Do you like to crack jokes and tell funny stories to your friends?
S. Do you think you usually worry too long after a distressing situation?

LAST QUESTIONS ARE ABOUT WEIGHT AND WEIGHT CONTROL

78. How tall are you?
_________ cm

79. How much do you weigh?
________kg

80. What has been your weigh at maximum (women: do not count pregnancies)
_______kg

81. How much did you weigh 12 months ago?
_______kg

82. How much did you weigh 5 years ago?
______kg

83. How much did you weigh when you were about 50 years old?
_____kg

84. How many times in your lifetime have you lost your weight over 5 kg on purpose?
   a. not once
   b. once
   c. 2 – 4 times
   d. 5 times or more

85. Finally, we ask you to measure your waist circumference with the enclosed measuring tape. Please measure it from the most narrow point or if you have difficulties finding it, in the middle of lowest part of the ribs and upper part of the hip bone. Please be at a standstill when you do the measuring.

My waist circumference is _______cm.

(PICTURE)

During the course of the study there might emerge some additional questions or need to focus some questions. Therefore we ask you to give us your e-mail-address and telephone number where we can reach you if necessary.

E-mail address

____

Telephone number

____

PLEASE CHECK THAT YOU HAVE ANSWERED TO ALL QUESTIONS.

WE THANK YOU WARMLY FOR YOUR ANSWERS!

When filling out the questionnaire some things might occur to you of which you want to tell us more about, or if you want to give some comments about the questionnaire, please write your notices here.

____
____
____
